# “It All Makes Us Feel Together”: Young People's Experiences of Virtual Group Music-Making During the COVID-19 Pandemic

**DOI:** 10.3389/fpsyg.2021.703892

**Published:** 2021-08-05

**Authors:** Maruša Levstek, Rubie Mai Barnby, Katherine L. Pocock, Robin Banerjee

**Affiliations:** School of Psychology, University of Sussex, Brighton, United Kingdom

**Keywords:** COVID-19, coping, virtual, group music-making, young people, mixed-methods, self-determination theory, school children

## Abstract

We know little about the psychological experiences of children and young people who have participated in virtual group music-making during the Coronavirus disease (COVID-19) pandemic. Adopting a mixed-methods design, we worked across three music education hubs in the UK, with a total 13 virtual music groups. These included a range of mainstream ensembles, inclusive ensembles targeting young people with special educational needs and/or disabilities, and inclusive music production spaces, targeting young people from lower socio-economic backgrounds. Reported progress in intra- and inter-personal psychological outcomes was investigated using quantitative and qualitative staff session reports, which were collected since before the pandemic (n1 for in-person sessions = 87, n2 for virtual sessions = 68), and surveys distributed to tutors, young people, and their parents during the first and second United Kingdom (UK) national lockdowns (n3 for qualitative responses = 240, n4 for quantitative responses = 96). Satisfaction of three basic psychological needs of self-determination theory and their relation to joint music-making in virtual spaces was also observed in real time by the researchers performing quantitative checklist observations on 16 separate occasions. Findings indicated that virtual music groups represented a meaningful psychological resource for the participating children and young people, especially considering the lack of opportunities offered by their schools and other extra-curricular activities. Through their participation with virtual group music-making activities, young people used music as a tool for self-expression and emotion management, restored lost musical identities and confidence, and preserved treasured social connections. Virtual alternatives to group music-making appear to indirectly nurture the sense of belongingness, mediated by supportive staff behaviors, but their direct connection, which has been widely reported for in-person group music-making experiences, has not been observed in virtual music groups.

## Introduction

Evidence suggests that engagement with the creative arts can have a positive impact on one's psychological well-being, reduce blood pressure and stress, as well as boost the immune system (Leckey, 2011; de Witte et al., 2020). The potential connection between arts and well-being has been particularly relevant since the outbreak of the Coronavirus disease (COVID-19), classified as a pandemic on 11th March 2020 by the World Health Organization (World Health Organisation, 2020). The COVID-19 pandemic, as a threat to public health, and due to social and economic effects of measures in place to slow its spread, e.g., national lockdowns and school or business closures, has had an enormous impact on mental health in the UK (e.g., Iob et al., 2020), and worldwide (e.g., Barzilay et al., 2020). Numerous studies have reported increased levels of stress, loneliness, and mental health difficulties (i.e., anxiety, depression, post-traumatic stress disorders) in adults (e.g., Rajkumar, 2020), as well as children and young people (e.g., Loades et al., 2020). Engagement with the arts as a way of collective coping with uncertainty and psychological distress due to the COVID-19 pandemic has been widely reported in the media on international scale. Examples include neighbors singing on balconies^1^, dancers performing outdoors^2^, photographers projecting their work on buildings^3^, and the British Broadcasting Corporation (BBC) organizing a virtual choir with over 15,000 people singing together^4^ In fact, engagement with music has been a particularly popular lockdown activity (e.g., Fink et al., 2021), which has attracted great attention from researchers around the world, such as through formation of the MUSICOVID international research network.^5^

International research has been illustrating the importance of music in times of spatial distancing, suggesting increased engagement, which has been a particularly valuable coping mechanism for many. For example, research in Spain suggests an increase in music use and music-related activities (e.g., singing, dancing) during lockdown, particularly for those not in active work (Cabedo-Mas et al., 2021). An international survey from Italy, Spain, and the United States of America has also observed greater engagement with music-making activities, such as playing an instrument, singing, or composing, when compared to before the pandemic (Mas-Herrero et al., 2020). International studies comparing music in relation to other domestic and leisurely activities have identified music as either the most useful coping mechanism during the first lockdown (Mas-Herrero et al., 2020), at least equally effective as other strategies in well-being support (e.g., mindfulness; Granot et al., 2021), or being ranked right behind keeping in touch, domestic chores, and TV in its importance during lockdown (Fink et al., 2021). Those identified as particularly emotionally vulnerable during lockdown (e.g., those living alone), or those impacted by the pandemic to a great extent (e.g., loss of work) had higher perceptions of musical behaviors' benefits (Martínez-Castilla et al., 2021), and engaged with it more, which was in turn associated with lower experiences of depressive symptoms (Mas-Herrero et al., 2020).

The demonstrated significance of musical engagement in times of spatial distancing is in line with existing research emphasizing the connection between musical engagement, psychological well-being, and coping; this has been observed via affect management, mood enhancement, sense of purpose, as well as stress and anxiety reduction (Daykin et al., 2018; de Witte et al., 2020). Individual factors predicting or mediating music's effectiveness for well-being support have also been identified, such as the ability to experience pleasure (Mas-Herrero et al., 2020), perceptions of music's importance (Martínez-Castilla et al., 2021), and the purpose of one's musical engagement (Fink et al., 2021). Fink and colleagues' (2021) cross-cultural study describes how those motivated to reduce negative emotions during the pandemic (e.g., loneliness, or stress) engaged in more solitary musical activities, while motivation to increase positive emotions was associated with a more social and aesthetic or spiritual experience of musical engagement, as a form of connecting with the self, as well as others.

Music has also been identified as a social determinant of health and well-being (e.g., Sheppard and Broughton, 2020), the significance of which has become particularly apparent in times of spatial distancing, a protective measure put in place in many countries in order to restrict the spread of the COVID-19 virus (World Health Organisation, 2020). The collective dimension of music-making has an additional, and sometimes amplifying impact on one's well-being. Research with therapeutic group interventions (e.g., Alcoholics Anonymous, or cognitive behavioral therapy groups) suggests that groups successfully establishing the sense of community and group identification also had more positive effects on participants' mental health (Steffens et al., 2021). In addition, research by (Iyer et al., 2009) suggests that the greater number of social group identities can protect against negative consequences of change on well-being. Choir singers reported higher levels of well-being than solo singers (Stewart and Lonsdale, 2016), and adults displayed higher levels of stress reduction hormones when their partners also engaged with music in everyday life (Wuttke-Linnemann et al., 2019). Social support, contact with like-minded others, and the sense of belonging to something greater than a sum of its parts were extremely important contributors to the sense of well-being in music groups (Stewart and Lonsdale, 2016).

The key factors associated with social bonding in musical contexts were shared goals and values, as well as perceptions of collective participation and being in a safe environment (e.g., Specker, 2014). Development of such experiences has been directly connected to group music-making (e.g., Hallam, 2015; Savage et al., 2020), through the process of sound co-creation and collective aesthetic experiences, also known as “sonic bonding” (Turino, 2008), as well as through synchronic movement. Interpersonal synchrony research suggests when individuals move in sync, they get on better, even in challenging contexts (Erfer and Ziv, 2006), are more likely to perform pro-social behaviors toward each other (Kokal et al., 2011; Stupacher et al., 2017), and have greater perceptions of social bonding with them (Tarr et al., 2016). Similar effects have been observed in adolescents and children (e.g., Kirschner and Tomasello, 2010).

However, spatial distancing regulations put in place in order to restrict the spread of the virus have also restricted collective engagement with music, with music groups having to halt their operation or resort to virtual alternatives. The collective aspect of music groups has been sorely missed in times of spatial distancing, and was identified as the most commonly and deeply perceived loss by choir members in Norway and Sweden (Theorell et al., 2020), and music groups in Belgium and The Netherlands (Onderdijk et al., 2021). The latter study indicated that during national lockdowns, live music-making in social settings decreased by 79% (Onderdijk et al., 2021). This has in turn been replaced by solitary and virtual musical engagement (Fink et al., 2021), with the same study by (Onderdijk et al., 2021) reporting an increase of 264% in virtual joint music-making since before the pandemic. Daffern et al. (2021) identified three virtual group music-making setup variations. “Multi-track” represents a mixed collection of pre-recorded solo contributions, “Live streamed” involves engagement via social media livestreams, and “Live tele-conferencing,” as the only variation allowing for interactive music-making, has been enabled via tele-conferencing softwares. Common video conferencing tools such as Zoom and Skype represented the majority of virtual platforms used (93%; Onderdijk et al., 2021), despite there being online platforms designed specifically for joint music making online (i.e., JamKazam, or Jamulus). However, those platforms do not allow for video or verbal interaction, emphasizing its value to the participants (Morgan-Ellis, 2021; Onderdijk et al., 2021).

Research with virtual music groups sheds light on the significant role collective music-making plays in such communities. Overall, studies conducted with virtual music groups have reported observed enhancements in mood, reduction in loneliness, and community preservation (Tarr et al., 2018; Daffern et al., 2021; Draper and Dingle, 2021; MacDonald et al., 2021; Morgan-Ellis, 2021; Onderdijk et al., 2021). Positive experiences were attributed to the sense of continuity (Daffern et al., 2021), pleasant experiences (Onderdijk et al., 2021), reconnection with memories of times before the pandemic (Morgan-Ellis, 2021), mood enhancement due to perceived creative agency, and increase in awareness of mood management strategies though creativity, as well as the sense of having a “music asylum” away from stress and worries (DeNora, 2013; MacDonald et al., 2021). The social dimension of virtual music groups was also recognized as important, with participants appreciating being able to maintain existing social connections (Daffern et al., 2021), reporting sense of connectedness and feeling of “being in the same boat” (MacDonald et al., 2021; Onderdijk et al., 2021). Some projects have even reported the development of new social relationships and virtual communities, and perceiving participation from home as more personal and intimate (Daffern et al., 2021; MacDonald et al., 2021; Morgan-Ellis, 2021).

However, in a quantitative study by Draper and Dingle (2021), group identification and basic psychological need satisfaction scores were not related to mental health scores. Furthermore, study reports have varied in terms of the perceived replication quality of virtual alternatives available. Research projects led by (Daffern et al., 2021), and (Onderdijk et al., 2021) concluded that participants recognized virtual alternatives as temporarily satisfactory, but not as a long-term substitute, especially in relation to the ability, or rather, inability, to make music together. (Daffern et al., 2021) participants did not think virtual choirs offered the same social benefits, reported the lack of peer musical support and feedback due to not being heard while making music, and reflected on the loss of “para- musical experiences” or the “magic” of the shared experience of making music together. However, they still recognized that virtual music groups fostered a sense of belongingness. (Onderdijk et al., 2021) observed that while pleasantness and connectedness were rated above average, perceptions of synchronization scored below average, showing a disassociation between synchronic movement and social bonding in the current context. Moreover, (Draper and Dingle, 2021) concluded group identification and basic psychological need satisfaction scores were lower for virtual music groups when compared to retrospectively allocated scores for face-to-face group music sessions, while still being relatively high, with means being above scale midpoints. Some research has observed the impact of group music-making loss in relation to group connectedness to a lesser extent, but these studies acknowledge that groups were not accessed by all in-person participants (MacDonald et al., 2021; Morgan-Ellis, 2021). While the connection between the loss of shared musical experiences in times of spatial distancing and group connectedness in virtual spaces is still not fully understood, we recognize none of the studies to date that are known to the authors researched young people's psychological experiences of virtual music group activities.

Despite the fact that children and young people have been particularly affected by the COVID-19 pandemic, there is a gap in research investigating whether virtual music groups managed to sustain and support their well-being in such challenging times as well. Research suggests that schoolchildren and adolescents have been disproportionately affected by the pandemic, especially in times of school closures, and unavailability of other extracurricular opportunities (Phelps and Sperry, 2020; Loades et al., 2021). The length and severity of lockdown restrictions, as well as the extent of marginalization (e.g., relating to ethnicity, low socio-economic background, special educational needs and/or disabilities) were also important contributors to the severity of the pandemic's impact on young people's social and emotional development in times of spatial distancing (Loades et al., 2021). According to the UK national survey, Mental Health for Children and Young People (MHCYP; National Health Service, 2020) conducted in 2017 and 2020 (during the COVID-19 pandemic, July), rates of credible mental health disorders amongst 5–16 year olds have increased by around 50%, estimated to reach 16% for the first year of the pandemic. Furthermore, decrements in social skills, confidence, and independence were observed in children and young people due to extended isolation and lack of social peer interaction (Demkowicz et al., 2020; Ofsted, 2020a,b). Increased loneliness observed in schoolchildren during the pandemic (Loades et al., 2020) could have a substantive effect on mood, development of social skills, self-identity, and its negative impact on mental health can be expected up to 9 years later (Loades et al., 2020; Orben et al., 2020).

**Figure 1:**
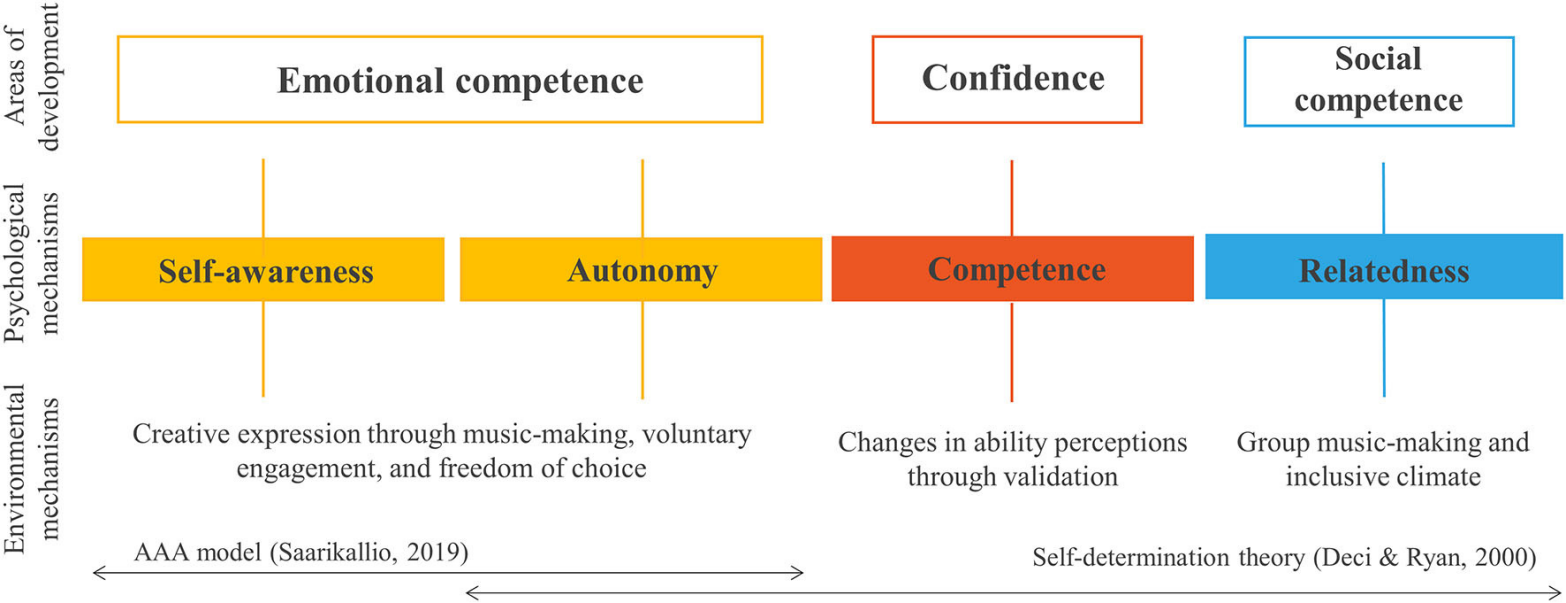
A model of psychological mechanisms of inclusive music-making, adopted from Levstek and Banerjee (2021).

Previous research with creative youth projects suggests that engagement with music-making could be of special value for the promotion of positive emotional and social competence and confidence in times of pandemic, as observed for adult virtual music groups. Engagement with creative arts in general, and music specifically, has been recognized as extremely beneficial for children and young people above and beyond their experiences at home and at school, in terms of psychological well-being, emotional competence, confidence, and social competence (e.g., Bungay and Vella-Burrows, 2013; Hallam, 2015; Russell et al., 2021). Psychological outcomes, and the underlying mechanisms, experienced by young people in inclusive music-making communities are represented in a model developed by Levstek and Banerjee (2021, see Figure 1), and informed by Self-determination theory (SDT; Deci and Ryan, 2000), and the Access-Awareness-Agency model (Saarikallio, 2019). Self-determination theory (SDT; Deci and Ryan, 2000) proposes that one's psychological well-being is dependent on the extent to which the surrounding environments satisfy basic psychological needs for autonomy, competence, and relatedness, while the Access-Awareness-Agency model describes music as a tool for expressing, accessing and becoming aware of one's psychological experiences, through which agency, ownership and greater emotional competence are developed.

Mixed-methods research by Levstek and Banerjee (2021) observed these processes in inclusive youth music spaces, identifying psychological outcomes of intra- and inter-personal development (emotional competence, confidence, and social competence), nurtured through mechanisms of self-awareness, autonomy, competence, and relatedness. Environmental mechanisms are also identified, with self-awareness and autonomy being determined to be highly dependent on creative expression through music-making, voluntary engagement, and freedom of choice. Validation and changes in ability perceptions through positive feedback were identified to nurture basic psychological need for competence, which in turn supports growth in confidence. Lastly, group music-making and inclusive climate are suggested to nurture one's sense of relatedness, which allows young people to feel comfortable socializing, and to develop in social competence. To our knowledge, only one research paper has focused on youth music-making in times of the pandemic, but this focused on music educators' dyadic teacher-student relationship experiences (de Bruin, 2021). The paper emphasizes the role music teachers have in creating an environment which fosters students' basic psychological needs for relatedness. However, further research is needed in order to better understand the role of virtual music groups in times of spatial distancing for schoolchildren and young people. The present study investigates the impact virtual youth music groups are having on intra- and inter-personal development, and how impactful the lack of group music-making was for its participants.

## The Present Study

The aim of the present work is to explore changes in young people's lived experiences of virtual group music sessions during the first and second national lockdown in the UK, when in person group music-making is not possible. In relation to the model described (Levstek and Banerjee, 2021; see Figure 1), we hypothesised that the route of social competence development, dependent on group music-making, will be impaired while other psychological mechanisms of self-expression, autonomy and competence will not be affected, and predicted that participating young people will report positive impact on emotional competence and confidence. The following hypotheses were proposed.

Hypothesis one: Young people will show greater development in intra-personal outcomes over their engagement in virtual group music-making, when compared to development in inter-personal outcomes.

Hypothesis two: Young people's needs for autonomy and competence will be more satisfied than the need for relatedness in virtual music sessions.

Hypothesis three: Virtual group music-making will be associated with feelings of relatedness, but only to the extent that they are associated with staff relatedness-supporting behaviors, in line with de Bruin (2021).

We worked with four music projects from Levstek and Banerjee's (2021) research, as well as nine other music groups delivering virtual sessions during the time period of two national lockdowns in the UK. Aspects of the model (Levstek and Banerjee, 2021) and their connections were explored through triangulation of three data sources, using a mixed-methods design. Quantitative and qualitative survey questions were administered to tutors and young people involved with the virtual music groups, as well as the young people's parents, exploring individual psychological experiences of the virtual music groups. This was further explored on the group level, and in comparison, to in-person sessions, with the use of “session reports” that the tutors in the music projects have been routinely completing following every group session since before the pandemic. Additionally, observations of virtual groups were conducted, quantifying staff behaviors in relation to psychological mechanisms, namely the satisfaction or thwarting of three basic psychological needs for autonomy, relatedness, and competence.

## Methods

### Participants

Purposive sampling was used. Specifically, the participants were staff members, young people and parents involved with three Music Education Hubs (local providers of music education in the southeast of the UK) that provided virtual group music-making opportunities during UK national lockdowns. Based on the nature of the sessions, the activities were categorized as: *Mainstream Ensembles*, representing virtual music group alternatives to in-person classical/traditional orchestras and ensembles for children and young people; *Music Spaces*, representing virtual music group alternatives to in-person music production and song-writing spaces, particularly targeting young people from lower socio-economic backgrounds; and *Inclusive Ensembles*, representing virtual music group alternatives to in-person inclusive ensembles, particularly targeting children and young people with special educational needs and/or disabilities. Those engaged with individual instrumental lessons or group instrumental lessons were not included in this research project.

As indicated in Table 1, we observed 12 staff members in online group contexts, which were attended by on average 5.04 young people per session observed. Furthermore, 240 qualitative and 96 quantitative survey entries relating to virtual group music-making for specific activities were obtained as secondary resources from Music Education Hubs' surveys for staff members, young people, and their parents. ^6^

**Table 1:** Participant demographics and data entry counts across project type activities.

**Project**	**Observations**				**Surveys**			**Session reports**
	**Staff members**		**Young people**		**Staff members**	**Young people**	**Parents**	
	**Total N (mean N per session)**	**Gender**	**Mean N per session**	**Age span**	**n responses**	**n responses**	**n responses**	**n responses**
Mainstream Ensembles (6)	8 (2.30)	F: 3	17.40	8 – 20	Qual: 10	Qual: 73	Qual: 28	–
		M: 5				Quan: 43	Quan: 26	
Music Spaces (3)	4 (2)	F: 2	2.83	12 – 20	Qual: 2	Qual: 3	–	Pre: 46
		M: 2				Quan: 7		Post: 57
Inclusive Ensembles (4)	–	–	–	–	Qual: 28	Qual: 62	Qual: 34	Pre: 41
						Quan: 15	Quan: 5	Post: 11
**Totals**	12	F: 5	5.04	8 – 20	Qual: 40	Qual: 138	Qual: 62	Pre: 87
		M: 7				Quan: 65	Quan: 31	Post: 68
					**Qual: 240; Quan: 96; Total: 336**			**Total: 155**

*N = number of participants, n = number of data entries; F = female or trans-female, M = male or trans-male, Qual = qualitative data entry, Quan = quantitative data entry. Responses are measured as the number of participants that completed each survey, and not the number of responses to each individual question (qualitative and quantitative surveys are treated separately, as they were not always distributed as one survey, however, Session Reports are an exception)*.

### Materials

#### Session Reports

Across the participating Music Education Hubs, music spaces and inclusive ensembles had been collecting session reports, completed by the music tutors following each group session, since before the pandemic (January 2019), and they continued to do so for the online group music sessions. The report includes two open-ended questions allowing staff members to provide an overview of session activities and note any details and significant events. The report template also contains five quantitative questions relating to group's personal and social experiences of the session. Regarding the group's social experiences, questions ask a staff member completing the report to reflect on the overall attitude of the young people toward each other, their overall attitude toward members of the wider community present at the venue during the session, and young people's communication with each other. These questions are answered on a scale from 1 (“*very bad”*) to 5 (“*very good”*), with 3 representing “*sufficient”*. There are also two questions asking how many of the young people displayed feelings of general well-being, and feelings of general self-worth or self-esteem, as a result of engagement with the particular session. These questions are answered on a scale from 1 (“*none of the young people or just a few”*) to 5 (“*all of the young people or nearly all”*), with 3 indicating “*about a half of the young people”*. The reports also record the setting of the session, whether in-person or virtual, and the number of young people present.

#### Surveys

We worked with music education hubs' internal evaluation teams to help gauge the psychological and musical impact that changes to virtual delivery mode had on staff members and young people involved. The staff surveys included open-ended questions exploring staff members' perceptions of the benefits and drawbacks of virtual group music-making in the context of the pandemic. Similar open-ended questions were also included in parent surveys, allowing parents to express their own perceptions of the virtual music sessions. Youth participation was also encouraged by adding separate questions in the parent surveys designed for parents to ask their children in order to record children's own views. In addition, quantitative ratings were elicited in order to tap into perceived changes in well-being, confidence, social skills, and feelings of calm and peace, from before to after engaging in the virtual music sessions: “*As a result of engaging with this virtual music-making session, my/my child's level of [outcome] has:,”* and response scale ranging from 1 (“*got very much worse”*) to 7 (“*got very much better”*), with the rating of 4 representing no change. The full list of all questions used in surveys can be seen in Supplementary Materials.

#### Observations

We adopted the Need-Relevant Instructor Behaviors Scale (Quested et al., 2018), developed for observing and recording exercise instructors' behaviors. The original scale was designed for recording the frequency of behaviors displayed by coaches and allocating intensity ratings of the impact this had on three basic psychological needs of participants. This scale was selected for two of its unique features in comparison to other SDT-based observation scales. Firstly, the checklist allows for recording behavior tallies as well as rating the perceived overall impact on participants' individual basic psychological needs for autonomy, competence, and relatedness. Secondly, besides containing separate categories for behaviors representing need-supporting, and need-thwarting types of behavior, the authors also created a dimension for need-indifferent behaviors, allowing observers to record neutral types of behaviors that are relevant to coaching.

During the planning and pilot visits of the virtual music sessions, the list of behaviors was rephrased and adjusted in order to be made relevant to music teachers' behaviors in a virtual environment and allow for observations of more than one teacher at once. Since the original list was carefully designed to cover a wide array of potential behaviors based on SDT literature (Quested et al., 2018), most items were kept or merged and rephrased or adjusted. Additionally, six items specific to musical and virtual context of teaching were added, and group music-making behaviors were also recorded, such as turn-taking, or playing alongside a recording or a teacher on mute. The final list contained 18 behavior items in total, reported in Supplementary Materials.

In order to adjust for live and busy observational context with multiple tutors, we separated sessions into 15-min chunks, with the first 12 mins dedicated to behavior frequency recordings, and the remaining 3 mins allowed for need impact scoring and preparation for the next chunk. Based on pilot testing, the 12-min frequency recording period was separated into six 2-min windows, allowing raters to record whether the behaviors were observed in the window or not. This resulted in behavior tallies ranging from 0, indicating no occurrence of the behavior at all, to 6, indicating the behavior occurred in every 2-min window of the chunk. Each 15-min chunk was observed by two raters independently, who each assigned frequency ratings for all need-supporting, need-indifferent, and need-thwarting behaviors. At the end of the 12-min observation period, the raters agreed on the need-supporting and need-thwarting impact scores for three basic psychological needs, allocating scores from 0 (“*no impact”*) to 3 (“*high impact”*). Those were then aggregated to represent an overall need impact score, ranging from −3 (“*high need-thwarting impact”*) to 3 (“h*igh need-supporting impact*”), with 0 representing “*no impact”*.^7^

All sessions were observed by Rater 1 (the first author) in combination with either Rater 2 or Rater 3 (the second and third authors). Inter-rater reliability for Rater 1 with either of the other raters was deemed satisfactory with the use of multiple calculation methods, namely Gwet's AC1 (0.72 for all items) and Brennan-Prediger's kappa (0.66 for all items) for a categorical format (agreement per each window), and intraclass correlation coefficient for a continuous format (number of windows recorded per chunk for each item; 0.86 for all items). See Supplementary Materials for further information regarding inter-rater reliability calculations and results.

### Procedure

#### Session Reports

All session reports were collected and shared with us by the Music Education Hubs. Staff members were encouraged to complete the reports routinely after a session took place, and to discuss their answers with the whole staff team present at the session. All young people's parents and young people involved with the sessions recorded in the reports were informed about the session reports, and all identifiable qualitative information was anonymised either in the process of completion of the report, or by us prior to the analysis. Sessions with only one young person present were not included in the analysis (*n* = 13). Out of the 142 remaining reports collected, 86 of the reports were completed for in-person sessions, 35 were collected during the first national lockdown or summer term of 2020, and 21 during the second national lockdown or autumn term of 2020. Quantitative data from all time periods were used in the analysis, while the qualitative analysis included open-ended questions completed for the virtual sessions.

#### Surveys

All surveys were collected and shared with us by the Music Education Hubs. Only those survey responses specifically referring to or collected during virtual group music sessions were included in the analysis. In total, 336 survey responses were collected, in particular 40 responses to staff surveys, 93 responses to parent surveys, and 203 responses from young people.^8^ Surveys were completed in relation to virtual group music sessions delivered during the first UK national lockdown or summer term of 2020 (ten groups surveyed, with 191 responses), or the second UK national lockdown or autumn term of 2020 (eight groups surveyed, with 145 responses).

#### Observations

All observation activities took place during the first UK national lockdown of the COVID-19 pandemic. All sessions attended had been running for at least a month prior to data collection. Piloting of the materials, and rater training have been conducted prior to observation data collection, described in the Supplementary Materials.

Twelve staff members delivering eight different virtual music activities were observed on two separate visits which were not consecutive. All staff members and participating young people were informed about the research project prior to data collection period and were given the dates of the researchers' visits. All staff members consented to be the subjects of observation, and verbal assent was obtained from the young people involved with the sessions (note that none of the young people were directly observed or had their individual behaviors recorded). There were on average 2.13 staff members present at each observed session, ranging from two to four, and raters noted which staff members were active and contributed to the behaviors recorded in each 15-min chunk. Sometimes the sessions would involve break-out rooms, a Zoom function allowing for allocation of participants into smaller groups with individual staff members in separate “rooms”. In such cases, the raters were kept together and able to move between the break-out rooms in order to observe all staff members for a similar amount of time.

In total, 16 1-h music sessions were observed, resulting in 64 15-min chunk observations, each coded by two separate raters. Some chunks were incomplete due to sessions ending before all six 2-min windows of the chunk could be completed, which were excluded from the analysis. As a result, 5 chunks were excluded, resulting in 59 full chunks being aggregated and analyzed.

#### Ethical Considerations

We obtained ethical approval from the University's ethical committee for secondary analysis of survey and session report data shared by the Music Education Hubs, as well as for the primary data collection involving virtual session observations.

### Analysis Plan

#### Quantitative Data Aggregation and Preliminary Analysis

Session report items were aggregated based on a confirmatory factor analysis (“lavaan” package, Rosseel, 2012). Two-factor solution was selected, representing “*Intra-personal outcomes”* variable (items regarding well-being and confidence; α = 0.81), and “*Inter-personal outcomes”* variable (items regarding communication skills and young people's attitude; α = 0.78). Based on this structure, survey questions representing change in well-being, calmness, and confidence were aggregated into the “*Intra-personal outcomes”* variable (α = 0.78), while the item representing change in social skills represented the “*Inter-personal outcome”* variable.^9^ Observation data was aggregated following a procedure suggested by Quested and colleagues (2018), which was adjusted in order to suit our modifications. ^10^ With each 15-min chunk as the observation data unit, final variables are: need-thwarting behaviors tally score; need-supporting behaviors tally score; need-indifferent behaviors tally score; group music-making behaviors tally score; and impact rating scores for autonomy, relatedness, and competence respectively. All tally variables were based on a scale from 0 (no occurrence) to 6 (occurs at least every 2 mins), and all impact rating score were based on the scale from −3 (high need-thwarting impact) to 3 (high need-supporting impact), with 0 as an indicator of no impact.

Descriptive statistics tables and correlation matrices were produced using RStudio (R Core Team, 2021) for all three quantitative datasets using the “finalfit” package (Harrison et al., 2020) and “rquery.cormat” function [Computing of Correlation Matrix, (n.d.)] respectively. Those tables are available in the Supplementary Materials.

#### Quantitative Analyses

Quantitative analyses were performed in RStudio (R Core Team, 2021), directly addressing our research hypotheses. In order to test the first hypothesis that young people will show greater development in intra-personal outcomes over their engagement in virtual group music-making, when compared to development in inter-personal outcomes, we performed mixed analyses of variance (ANOVA) on session report and survey data using “lmerTest” and “BayesFactor” packages (Kuznetsova et al., 2017; Morey and Rouder, 2018), followed by appropriate *post-hoc* tests.

For the ANOVA on session report data, a random intercept model investigated the main effects of the between-subject variables, session type (“inclusive ensembles” vs. “music spaces”), and setting (“in-person” vs. “virtual”), as well as the within-subjects variable, outcome category (“intra-personal” vs. “inter-personal”), and its interaction with the setting variable. For the ANOVA on surveys, a random intercept model investigated the main effects of the between-subjects variable, session type (“mainstream ensembles,” “inclusive ensembles,” “music spaces”),^11^ and within-subject variable, outcome category (“intra-personal” vs. “inter-personal”). Parameters and their 95% confidence intervals were estimated using a Bayesian inference method with “rstanarm” package, an R interface to the Stan C++ library for Bayesian statistical inference with the Markov Chain Monte Carlo sampling (Brilleman et al., 2018; Goodrich et al., 2020). Partial omega squared values (Olejnik and Algina, 2003) were calculated for individual items (“effectsize” package, Ben-Shachar et al., 2020), as well as the variance inflation factor (VIF; “performance,” Lüdecke et al., 2020). For session repots, change in score from in-person to virtual session setting was further investigated for the separate outcome variables with independent t-test analyses (intra- and inter-personal outcomes; “stats” package; R Core Team, 2021), Cohen's d calculations (“effectsize”), and Bayesian superiority analyses (“baymedr” package; Linde and van Ravenzwaaij, 2019; van Ravenzwaaij et al., 2019). ^12^ Lastly, intra- and inter-personal outcome variables of session report (virtual setting only) and survey data were compared against the scales' mid-points representing middle ground or no change. Selected values of reference were “three” for session reports, representing the middle ground on a group-level (either “*about a half of the young people”* or “*sufficient”*), and “four” for survey data, indicating “*no change”*.^13^ This was performed utilizing one-sample *t*-tests (“stats” package), Bayesian estimations of credible intervals^14^ (“BayesianFirstAid” package, Bååth, 2013), and Cohen's d effect sizes.

In order to test the second hypothesis that young people's needs for autonomy and competence will be more satisfied than needs for relatedness in virtual music sessions, we performed a mixed analysis of variance (ANOVA) as outlined above. A random intercept model investigated main effects of the between-subject variable, session type (“mainstream ensembles” vs. “music spaces”), and within-subject variable, basic psychological need type (autonomy, competence, relatedness)^15^. Individual basic psychological need impact scores were compared against the value of “zero,”^16^ representing no impact, following the same one-sample test procedure outlined above.

Lastly, to test the hypothesis that virtual group music-making will be associated with feelings of relatedness, but only *via* staff need-supporting behaviors, we performed a mediation analysis with the mean group music-making tally variable as the predictor, staff need-supporting behaviors tally variable as the mediator, and relatedness impact score as the dependent variable (“lavaan” package). As need-supporting staff behaviors referred to autonomy-, competence-, and relatedness-supporting behaviors, we performed the corresponding mediation analyses with autonomy impact ratings, and competence impact ratings as dependent variables to evaluate whether the pattern was specific to relatedness or generalised across all basic needs. All standard errors and test statistics were calculated using bootstrapping.

#### Qualitative Analysis

Responses to open-ended questions in the surveys and session reports were analyzed with NVivo12 software, using the thematic analysis approach described by Braun and Clarke (2006). An inductive and semantic approach was adopted in coding, allowing for unbiased exploration of tutors', young people's and parents' experiences of virtual music groups, separate from our research hypotheses. Following this, in line with our hypotheses and the model of psychological mechanisms of inclusive music-making (Levstek and Banerjee, 2021), the themes that had emerged were categorized into superordinate categories of “*Intra-personal experiences”* and “*Inter-personal experiences”*. Results were discussed with and approved by music tutors and key stakeholders at stakeholder meetings and a staff conference.

## Results

An integrated account of the quantitative and qualitative results is presented below, in relation to each of the three research hypotheses. Additionally, session report, survey, and observation ANOVA results are presented in Table 2, while all qualitative analysis themes are collected in Table 3. All effect sizes reported are partial omega squared values (Olejnik and Algina, 2003) and interpreted following Field (2013), and all Cohen's d values were interpreted according to Sawilowsky (2009).

**Table 2:** Fixed-effects ANOVA results for session report, survey, and observation scores.

	**Sum of Squares**	**Mean Square**	**Df (numerator)**	**Df (denominator)**	***F*-value**	***P*-value**	**Partial Ω^2^**	**Partial Ω^2^ 95% CI [LL, UL]**	**VIF**
**Session report ANOVA**									
Session type	0.04	0.04	1	139	0.23	0.635	<0.001	[0.00, 0.00]	1.09
Outcome type	0.01	0.01	1	140	0.03	0.854	<0.001	[0.00, 0.00]	1.65
Setting	1.04	1.04	1	139	5.50	0.020 *	0.03	[0.00, 0.09]	1.45
Setting:Outcome	2.76	2.76	1	140	14.64	<0.001 ***	0.09	[0.03, 0.17]	2.01
**Survey ANOVA**									
Session type	0.03	0.02	2	77.26	0.03	0.968	−0.02	[0.00, 0.00]	1.00
Outcome type	8.61	8.61	1	104.67	17.16	<0.001***	0.13	[0.05, 0.24]	1.00
**Observations ANOVA**									
Session type	0.08	0.08	1	57	0.22	0.645	−0.01	[0.00, 0.00]	1.00
Need type	7.38	3.69	2	116	9.92	<0.001 ***	0.13	[0.04, 0.22]	1.00

*Df = degrees of freedom; LL and UL = lower limit and the upper limit of the partial Ω^2^ confidence interval = respectively; VIF = variance inflation factor*.

**Table 3:** Identified themes by supra-ordinate categories and their hypothesis relevance.

**Supra-ordinate category**	**Theme**	**Hypothesis relevance**
(1) Intra-personal experiences	(1.1) Lockdown musical lifeline	Hypothesis 1
	(1.2) Confidence restoration	Hypothesis 1
	(1.3) Virtual autonomy	Hypothesis 2
(2) Inter-personal experiences	(2.1) Virtual social skills	Hypothesis 1
	(2.2) Virtual communities	Hypothesis 2
	(2.3) Group validation	Hypothesis 2
	(2.4) Bonding through virtual group music-making	Hypothesis 3

### Testing Hypothesis 1: “*The Difference to [Their-Child's] Mood Each Week Has Been Noticeable” [Parent]*

In order to test the first hypothesis that young people will show greater development in intra-personal outcomes over their engagement in virtual group music-making, when compared to development in inter-personal outcomes, we performed two mixed analyses of variance with session report scores (session type, setting, and outcome type as predictors), and survey scores (session type, and outcome type as predictors).

**Figure 2:**
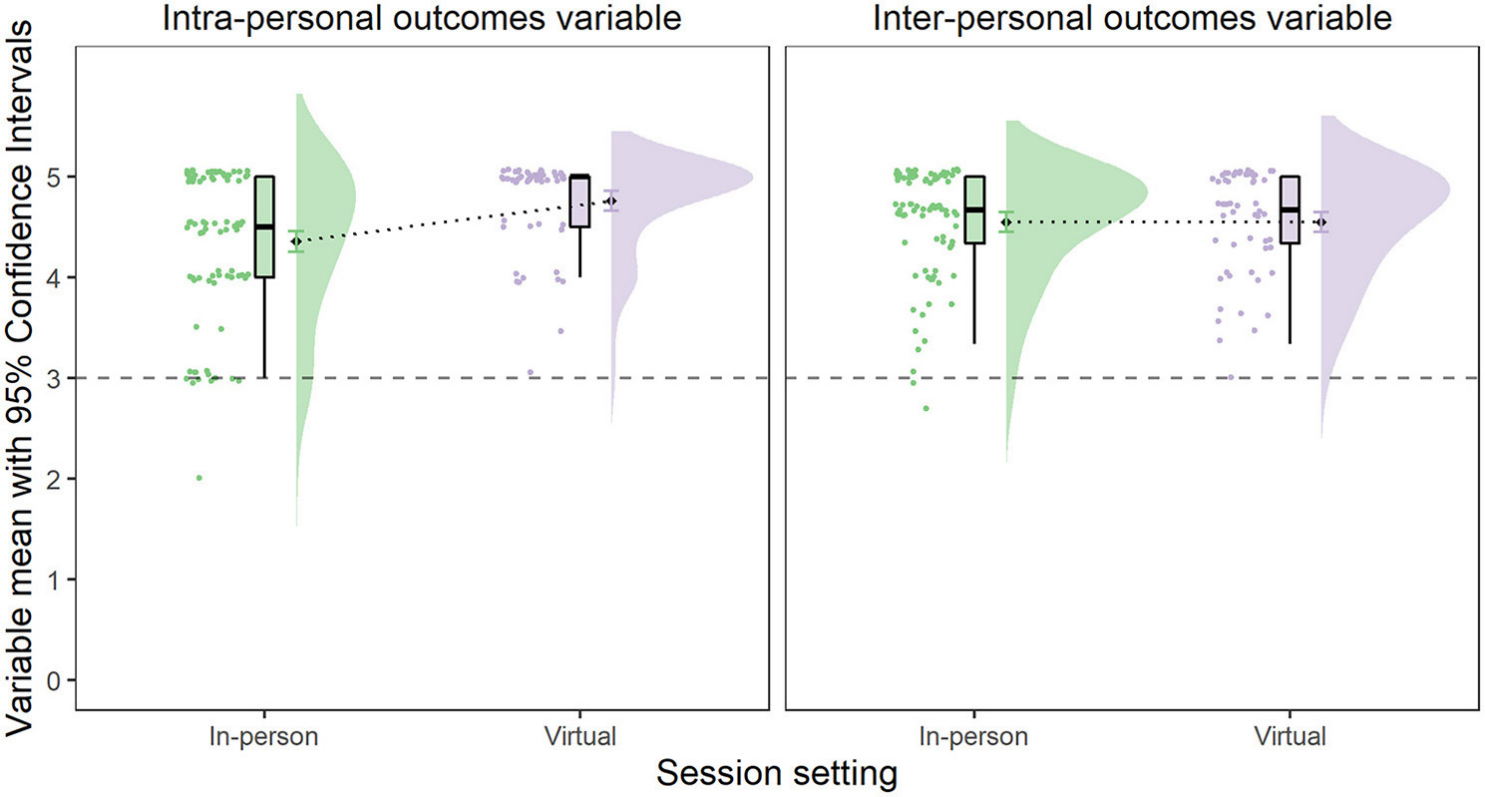
Raincloud plots of change in intra-personal and inter-personal session report variable scores when session setting changed from in-person to virtual.

As demonstrated in Table 2, session type yielded a non-significant fixed effect with very small effect sizes for both datasets. For the session report ANOVA, the main effect of outcome type was very small and non-significant, while the effect of session setting was significant and small in effect. Overall, virtual setting scores (*M*= 4.65, *SD* = 0.51) were higher than in-person session report scores (*M* = 4.45, *SD* = 0.66; *b* = 0.42, 95%*CI* [0.21, 0.63]). The interaction between outcome type and session setting was significant with medium effect size, and the Bayesian model variation with the interaction-only effect yielded the highest Bayes Factor (*BF* = 1727.68). As demonstrated in Figure 2, scores for the intra-personal outcome dimension increased as a result of transition from in-person (*M* = 4.35, *SD* = 0.74) to virtual music setting (*M* = 4.76, *SD* = 0.46; *t*_(140)_ = 3.66, *p* < 0.001, *d* = 0.66, 95%*CI* [0.34, 1.04], *BF* = 138.71). On the other hand, inter-personal outcome dimension scores for in-person (*M* = 4.55, *SD* = 0.55) and virtual music sessions (*M* = 4.55, *SD* = 0.53) did not differ {*t*_(140)_ = 0.01, *p* = 0.99, *d* = 0.00, 95%*CI* [−0.33, 0.34], 1/*BF* = 1/0.19 = 5.26}. Still, both intra- and inter-personal variable means for virtual session setting were greater than the neutral value of three (for intra-personal: *t*_(55)_ = 16.52, *p* < 0.001, *d* = 2.21, probability of mean being > 3 is > 0.99; and intra-personal: *t*_(55)_ = 11.21, *p* < 0.001, *d* = 1.50, probability of mean being > 3 is > 0.99). This indicates staff members still noticed positive changes in both dimensions overall.

**Figure 3:**
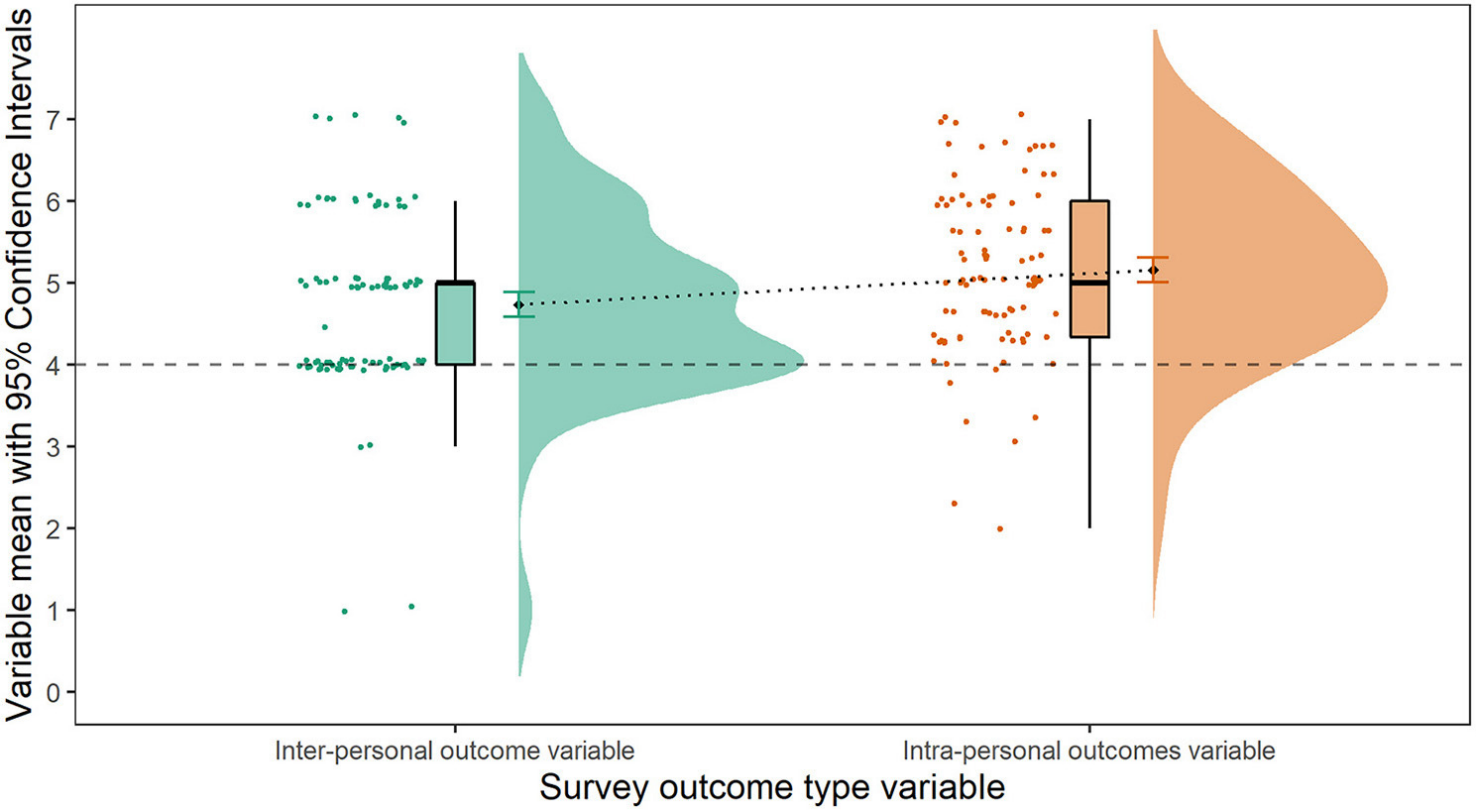
Raincloud plots of the difference between inter-personal and intra-personal survey variable scores.

For the survey ANOVA, the main effect of outcome type was significant and medium, and its relevance was confirmed by the highest Bayes Factor for the model with this predictor only (*BF* = 264.67 ± 0.74%). As demonstrated in Figure 3, intra-personal survey scores (*M* = 5.16, *SD* = 1.01) were higher than inter-personal survey scores (*M* = 4.73, *SD* = 1.08, *b* = −0.43, 95%*CI* [−0.64, −0.22]), and greater than the value of four, which represents no change [*t*_(95)_ = 3.97, *p* < 0.001, *d* = 0.41, probability of mean being > 4 is > 0.99]. However, the inter-personal survey score mean was not different from the value representing no perceived change in inter-personal constructs as a result of virtual group music-making [*t*_(95)_ = −0.14, *p* = 0.89, *b* = −0.01, probability of mean being more than 4 is 0.42]. Respondent type when added to the ANOVA model was non-significant, suggesting that young people and parents were giving similar scores overall, *F*_(1,186.53)_ = 2.39, *p* = 0.12, Ω^2^ *(partial)* = 7.32e−03 [0.00, 0.04]. The outcome type estimate did not differ when respondent type was included in the model [*b* = −0.42, *t*(100.64) = −4.23, *p* < 0.001], and when not [*b* = −0.42, *t*(104.67) = −4.14, *p* < 0.001].

Qualitative analysis themes “*Lockdown musical lifeline*,” “*Confidence restoration*,” and “*Virtual social skills”* explore differences in young people's intra- and inter-personal developments further. “*Lockdown musical lifeline”* theme describes how virtual music sessions were an important source of well-being during lockdown, “*a musical lifeline*” *[parent]*, and, for many, the only continued activity available. In the context of lockdown and unavailability of many other activities, these sessions worked as a distraction from the pandemic, gave young people something to look forward to, as well as introduced a sense of routine and “old normality” to their lockdown life. A staff member in a session report mentions a participant who “*doesn't do any other activities and barely leaves [their] room all week—this is reportedly the one activity that [they] take part in, and [they] look forward to it” [session report]*. Another parent notices how “*the consistency and stability that it [virtual music activity] has provided has been enormous, and the difference to [their -child's] mood each week has been noticeable” [parent]*. Additionally, young people used music as a way to connect with themselves and express how they are feeling about the current climate, visible in incorporating relevant motives and themes in their music work (i.e., writing lyrics about isolation).

The importance of music sessions in the context of the pandemic and lack of other activities was also recognized as important in the “*Confidence restoration”* theme, with an example of a parent reflecting how a particular music group “*has given [the child] some confidence that [they—the child] can do it as I [the parent] think was lost a little in home schooling” [parent]*. Growth in confidence was also attributed to young people being reminded they “*can still play as well as before*” *[young person]*, and to participation from one's home environment, from where some felt more comfortable to engage.

The theme “*Virtual social skills”* describes how young people also developed in inter-personal skills, particularly in virtual communication. Virtual communication included speaking unmuted, but also participating via chat function or Zoom reactions buttons. This, in fact, provided “*more options available to young people who are non-verbal / not comfortable with communicating to a large group” [staff member]*. Furthermore, staff members noticed “*the increase in respect for others and leaders from the young people [… which] could be down to the mute function” [staff member]*. However, there were fewer opportunities for social contact in virtual sessions, *and “not many accidental chats or usual flashes of personality” [staff member]*. Still, social interaction was often facilitated by mutual interest in music and participating from home environment, creating “*opportunities to share things that are personal to them in their homes*, e.g., *showing others their pets on the screen, Christmas decorations etc*.” *[staff member]*.

### Testing Hypothesis 2: “*It Can't Replace Actual Contact Teaching and Having the Experience With Someone” [Staff Member]*

Further, we performed a mixed analysis of variance (ANOVA) with the basic psychological need impact scores derived from observations of the virtual sessions (with session type and need type as predictors), in order to address the hypothesis that in virtual music sessions, young people's needs for autonomy and competence will be more satisfied than the need for relatedness.

**Figure 4:**
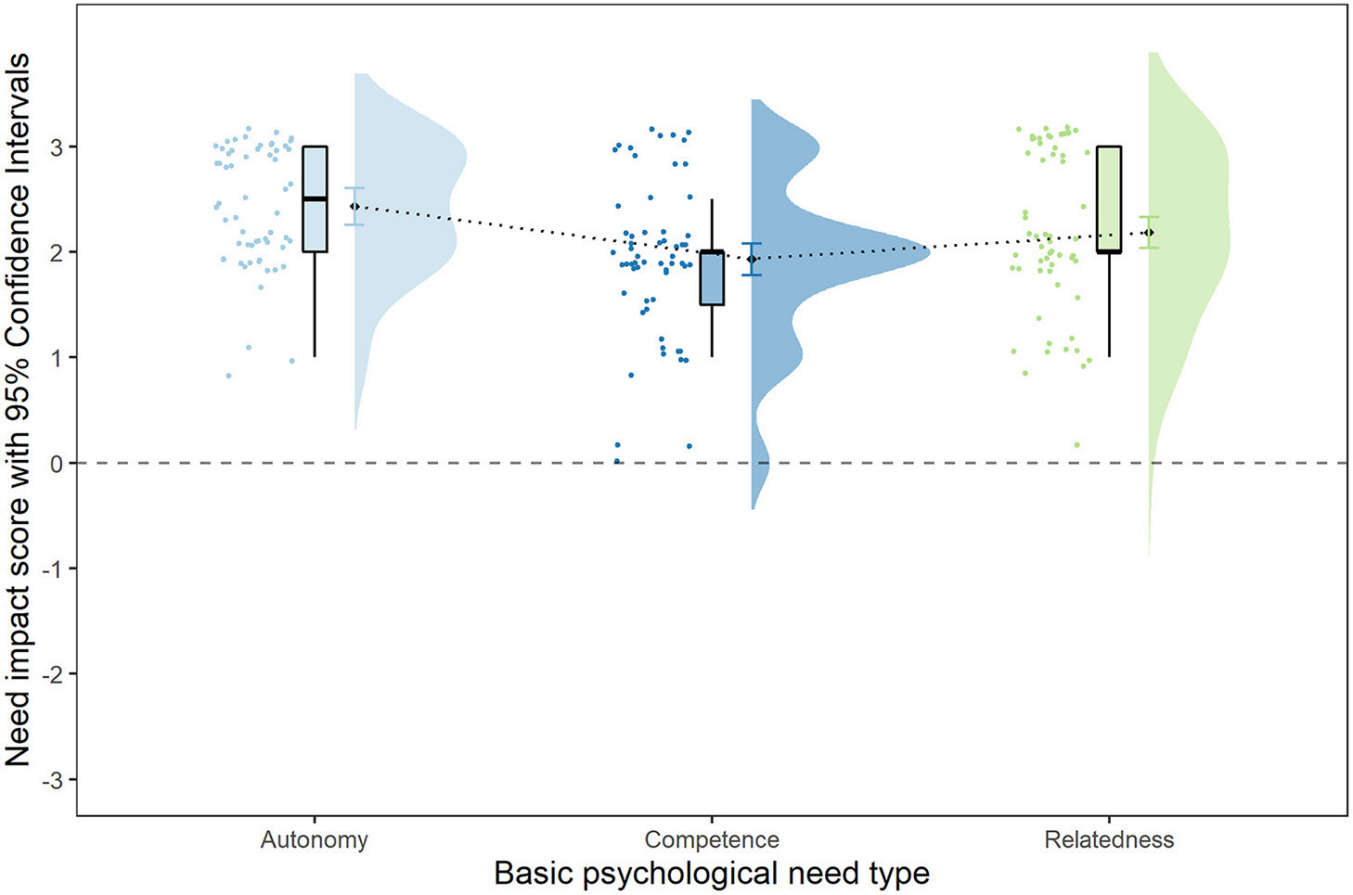
Raincloud plots of the difference between observation impact ratings for the basic psychological needs autonomy, competence, and relatedness.

As represented in Table 2, session type was non-significant and very small in effect, while need type was significant with medium effect size. Bayesian model variation comparison indicated the greatest factor for the model including need type only (*BF* = 799.25). *Post-hoc* comparisons indicated that when mean rating scores for autonomy and competence were compared against relatedness score, those did not differ [*b* = −0.00, *t*_(116)_ = −0.04, *p* = 0.97]. However, this was due to autonomy (*M* = 2.43, *SD* = 0.58) scores being significantly greater than competence scores [*M* = 1.93, *SD* = 0.75, *b* = 0.25, *t*_(116)_ = 4.45, *p* < 0.001], while relatedness mean scores were between the two (*M* = 2.19, *SD* = 0.77), as visible represented in Figure 4. All individual rate scores were significantly greater than the value of zero, representing no impact, with probability > 0.99 [autonomy: *t*_(58)_ = 12.44, *p* < 0.001, *b* = 1.62; competence: *t*_(58)_ = 4.42, *p* < 0.001 *, b* = 0.58; relatedness: *t*_(58)_ = 6.84, *p* < 0.001, *b* = 0.89]. When observation context variables were added to the model, need type comparison estimates did not change, and their main effects were non-significant: observation chunk, *F*_(3,52)_ = 0.23, *p* = 0.88; observation repetition, *F*_(1,52)_ = 0.02, *p* = 0.89; and rater pair, *F*_(1,52)_ = 0.25, *p* = 0.62.

Qualitative themes “*Virtual autonomy*,” “*Virtual communities*,” and “*Group validation”* explore development of the three basic psychological needs in virtual environments further. Virtual setting features played a role in nurturing of young people's sense of autonomy and ownership of their learning, represented by the theme of “*Virtual autonomy”*. “*Being able to control the camera (off/on/where it points/what you show it) and the use of chat gave the young people a lot of control over their virtual environment” [staff member]*. This was further nurtured by being able to participate from one's own home, having to take greater “*responsibility for their own materials [and] their own note-taking” [staff member]*, and with having “*more time on their hands, a lot of children also tried extra pieces on their own and developed greater ownership of their work” [staff member]*. There was also evidence of youth-led teaching approach, with young people and in some cases, parents, being able to contribute to session and activity planning, and getting “*some insight into the content of the lessons” [young person]*.

Further, the theme “*Group validation”* describes how competence supporting behaviors, such as validation and positive feedback, appear to have been highly dependent on opportunities for music-making, but only when staff members were able “*to hear everyone play—so all musical contributions were heard and validated” [session report]*. Over time, there were descriptions of young people becoming “*much more supportive of each other as a group” [session report]*, and “*began giving feedback which felt like a significant progression” [session report]*. Peer feedback was sometimes verbal, but “*many also used the chat function, to make positive contributions and feedback” [session report]*. However, due to latency and connectivity difficulties groups were “*to be muted when playing as a whole group” [staff member]*, meaning there were fewer opportunities to hear others play and provide feedback, unless “*playing on their own (when they unmuted themselves)” [staff member]*.

Lastly, the theme “*Virtual communities”* emphasizes that the sense of being part of a music group was present even when music groups met virtually. Social contact “with the outside world” enabled through these music sessions was particularly important as “*a lot of things they [young people] had from school were either pre-recorded or set work that wasn't delivered live by a teacher” [staff member]*, and parents noted that “*many children have become even more isolated since COVID” [parent]*. When young people were asked about what they enjoyed the most during the online rehearsals, answers such as “*seeing everyone*,” “*staying in touch*,” and “*being able to see everyone—I miss them*” *[three respective young people]* were common. Social contact appears to be incredibly important to young people as many wished for “*more of an opportunity to talk with others” [young person]*, and staff recognize “*there was quite a bit of talking about things unrelated to music” [session report]*, especially when in break-out rooms. New virtual communities were formed, as “*online [setting] is good in that it crosses barriers between students in terms of location, and to some extent level of achievement” [staff member]*. There appears to have been a generally inclusive climate toward the newcomers, with young people mentioning “*[it] was really nice to be so social with people [they] already knew and newer people as well” [young person]*, and enjoyed being able “*to work with people that [they] wouldn't have usually” [young person]*. Even in newly established groups, young people were eventually showing more ease to interact and opening up about personal topics. However, the inter-personal dimension of music groups was much harder to replicate, as summarized by a staff member below.

“*I think in some cases it has allowed more focus and created a space to feel more creative in but also has allowed a confidence to develop and take ownership of the learning. However, as great as this medium is and by creating opportunity to see people in different settings and areas and allowing to continue learning, it can't replace actual contact teaching and having the experience with someone.” [Staff member.]*

### Testing Hypothesis 3: “*As Good as it Can be Given the Current Virtual Limitations” [Young Person]*

**Figure 5:**
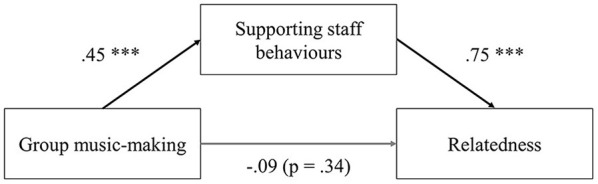
Test statistics for the relationship between group music-making and satisfaction of basic psychological need for relatedness, mediated by supporting staff behaviors, ***indicates statistical significance *p* < .001.

Lastly, in order to address the final research hypothesis that virtual group music-making will be associated with feelings of relatedness, but only *via* staff need-supporting behaviors, we built a mediation model with relatedness basic psychological need impact score as the outcome, mean virtual group music-making tally variable as the predictor, and need-supporting staff behavior tally variable as the mediator. Significant Sobel test indicated that supporting staff behaviors mediated the relationship between group music-making and perceived relatedness support, *b* = 0.11, β = 0.23, *SE* = 0.05, *p* = 0.04. As visible in Figure 5, the indirect relationship between virtual group music-making and relatedness need scores was mediated via need-supporting staff behaviors, which was significant, *b* = 0.15, β = 0.33, *SE* = 0.05*, p* = 0.001, while the direct effect of virtual group music-making on perceived relatedness support was not. It should be noted that there was no such mediation effect of need-supporting staff behaviors on rated support of autonomy (*b* = 0.05, β = 0.14, *SE* = 0.04, *p* = 0.23), or competence (*b* = 0.08, β = 0.18, *SE* = 0.07, *p* = 0.21). All test statistics reported are based on bootstrapping.

The theme “*Bonding through virtual group music-making”* addresses this hypothesis further. Playing together in time was not supported by the virtual setting due to latency and sound quality challenges, meaning participants had to resort to alternative approaches. Most common virtual group music-making approaches were playing along to the pre-recorded backing tracks or someone playing “unmuted” (meaning with sound on Zoom) while everyone else was “on mute” (meaning with no sound on Zoom), or turn-taking in “unmuting” (meaning activating sound on Zoom) and playing. Some groups also organized end-of-term performances in the form of a group video consisting of individuals' videos and audios pieced together. While virtual sessions were recognized as a fair substitute, “*as good as it can be given the current virtual limitations” [young person]*, the hope for in-person music-making sessions to become available again was ever-present. There was a general consensus that “*it [group music-making alternatives] didn't create the sense of real time interaction that group music-making allows, and -no matter what we were able to offer—it wasn't a profound group musical experience” [staff member]*. The lack of playing together impacted young people's learning, as well as their social experiences. Many reflected how the inability to play together “*loses the social side of music-making and the sense of ensemble” [staff member]*, and was described as “*the biggest drawback in that this is probably the most important aspect of social music making” [staff member]*. However, participants mention group music-making alternatives “*kept alive the social aspect of playing an instrument and being in an orchestra” [young person]*. A young person describes how the alternatives allowed them ”*to feel like we were in a band and playing together even though we could only hear our own contributions” [young person]*, and another young person mentions how “*it all makes us feel together, even when we are not together” [young person]*.

## Discussion

Young people's psychological experiences of virtual music groups were investigated in relation to a model of psychological mechanisms of inclusive music-making (Levstek and Banerjee, 2021; see Figure 1). The type of session activity was non-significant in all analyses of variance, indicating mainstream ensembles, inclusive ensembles targeting young people with special educational needs and/or disabilities, and music production spaces aimed at those from lower socio-economic backgrounds did not differ in perceived developments in inter- and intra-personal outcome areas, or in observed basic psychological need satisfaction. All quantitative scores were above scale mid-points (except young people and parent survey ratings for developments in social skills), illustrating a generally positive picture in terms of perceived psychological experiences of virtual group music sessions for children and young people. The analyses supported our first hypothesis that young people will show greater development in intra-personal outcomes than in inter-personal outcomes over their engagement in virtual group music-making. The second hypothesis that young people's needs for autonomy and competence will be more satisfied than needs for relatedness in virtual music sessions was only partially supported. The observed impact was the greatest for the basic psychological need for autonomy, followed by relatedness, and lastly, competence. Finally, mediation analysis supported our final hypothesis, demonstrating that relatedness support scores were indirectly related to virtual group music-making. The relationship between the two was mediated by need-supportive staff behaviors.

### Intra-Personal Experiences of Virtual Music Groups

Our results provide consistent evidence for the positive impact virtual engagement with music has on young people's intra-personal experiences in times of spatial distancing. The route of emotional competence development as represented in the model by Levstek and Banerjee (2021; see Figure 1), and based on Saarikallio's (2019) Access-Awareness-Agency model, was also recognized in descriptions of young people employing music as a tool of creative expression of lockdown hardship. The Access-Awareness-Agency model (Saarikallio, 2019) regards music as a medium through which one can access, understand, and own emotional experiences, which is exactly what the descriptions of young people managing their emotions via creative incorporation of themes of isolation described. As also observed in existing research with adult virtual music groups (e.g., Daffern et al., 2021; MacDonald et al., 2021), these opportunities represented a continuation of the old routine, gave young people something to look forward to, and served as a distraction from reality of the “new normal,” or in words of DeNora (2013), as a musical asylum. Out of the three basic psychological needs of Self-determination theory, which proposes that if all three needs^17^ are satisfied by a particular environment, one will experience positive psychological well-being (Deci and Ryan, 2000), autonomy was rated as the highest in terms of the positive impact staff behaviors had on its satisfaction. Indeed, young people described feeling they can contribute to the construction of the sessions and activities planning, which was labeled as a “youth-led” approach in the model by Levstek and Banerjee (2021), and recognized as valuable in autonomy support by the existing SDT research (Skinner and Belmont, 1993; Assor et al., 2002; Reeve, 2006). This appears to be further supported by virtual engagement, allowing control and choice over virtual features (e.g., camera, chat function).

Confidence restoration, rather than development, was described by our participants, affirming the devastating impact of the pandemic and isolation on young people's psychological health emphasized by previous research (Demkowicz et al., 2020; Ofsted, 2020a,b). Young participants reflect that by participating in virtual music groups, they were reminded of their identity as musicians, which allowed them to regain some of the confidence lost. Indeed, engagement with activities differentiating children and young people from others becomes crucial for self-identity formation around middle childhood (Harter, 1999, as cited in Hargreaves et al., 2002), and participation in formal musical education was recognized as crucial for assimilation with “musical identities” (Lamont, 2002). Furthermore, discrepancies in one's self-image and actual behavior can result in psychological distress, often manifested in lowered self-esteem (Rogers, 1961, as cited in Hargreaves et al., 2002), such as when young musicians with developed musical identities were unable to participate in formal music-making setting before virtual alternatives were available. Therefore, consistency in one's perceptions of the self and one's action were a crucial part of confidence restoration described by our participants.

Furthermore, in Levstek and Banerjee's (2021) model, growth in confidence was mapped as highly dependent on environmental support for the basic psychological need competence. Although still rated as positive, staff competence support was rated as the lowest of the three in the researchers' observations. This could be explained by reported reduction in opportunities for constructive feedback in virtual environments, which was established as one of teacher behaviors supporting students' sense of competence in virtual learning environments (amongst structure and guidance, but see Chiu, 2021 for further information). Staff members reflect that providing constructive feedback was only possible when they were able to hear young people play, which was less available in a virtual context as most music-making occurred in private, while being “on mute” (meaning with no sound on Zoom). This deprivation of feedback opportunities was also mentioned in research by (Daffern et al., 2021), and the importance of music-making should therefore be recognized in relation to competence building in the model by Levstek and Banerjee (2021).

### Inter-Personal Experiences of Virtual Music Groups

The inability to replicate the experience of making music together in virtual setting presented a unique opportunity for exploration of its role and meaning to musical communities. Those accessing group music-making alternatives perceived their virtual inter-personal experiences as satisfactory considering the restrictions but expressed a sense of loss of social connections through the lack of socializing opportunities and joint music-making. This has been identified as an important mechanism for the development of relatedness and consequently growth in social skills, as suggested by Levstek and Banerjee (2021). While on the individual level, young people and their parents did not perceive engagement with sessions had a positive impact development of social skills, staff members disagreed. Session report scores for inter-personal dimension were greater than the scale mid-point, indicating that tutors did observe displays of communication skills, and positive attitudes. Additionally, impact on the basic psychological need relatedness support was rated positively, suggesting belongingness was also nurtured in those music groups. A mere social contact “with the outside world” and a familiar group that these music sessions represented was important and recognized as enough to re-establish the sense of pre-existing music communities, while formation of new virtual communities was also observed, welcoming those unable to participate in-person. This was in line with the narrative of already published research with adult music communities in times of spatial distancing (Daffern et al., 2021; MacDonald et al., 2021; Morgan-Ellis, 2021; Onderdijk et al., 2021).

Despite this, there was a perception of the lack of opportunities for socializing, “*accidental chats or usual flashes of personality*,” as one of the staff members put it, exacerbated by the loss of group music-making. Group music-making as in-person was impaired in virtual context, and two models of virtual group music-making identified by (Daffern et al., 2021) were also described by our participants. “Multi-track,” a mix of pre-recorded individual contributions was an occasional occurrence, mostly in the context of the more common model of “live tele-conferencing,” in our case interactive music-making via Zoom software, popular among many music groups (Onderdijk et al., 2021). Further, we identified two sub-types of live virtual group music-making, “together on mute,” playing alongside a backing track or one unmuted person, and “turn-taking solo,” playing unmuted in front of others. This sense of loss and attempt to re-create the experience of communal music-making reflects the importance of shared musical experiences, and their value to its participants. Loss of “*the sense of real time interaction that group music-making allows” [staff member]* reminds us of the experience of “sonic bonding,” as described by Turino (2008), or the “magic” of shared aesthetic experience of making music together, the absence of which was also discussed by virtual choir members participating in (Daffern et al., 2021) study. Research from a range of disciplines consistently indicates that group music-making plays a crucial role in social bonding (Specker, 2017), and yet, even though missed, the sense of togetherness was still reported and even formation of new communities was recognized in our, as well as other, virtual group music-making communities. Similar observations have been made by (Onderdijk et al., 2021) research with adult choirs members, who rated perceived virtual connectedness above average, but this was not the case for perceptions of enabled synchronization, although the link between the two has been widely established in past research (e.g., Tarr et al., 2014, 2016). Specker describes the importance of “*practices of engaging with one another through singing, by way of a common goal, shared values, a safe environment, community interaction, and social infrastructure*” (Specker, 2014, p. 87). Our research with virtual music-making settings shows that these practices may be preserved even in the temporary absence of joint engagement with music in person. Through engagement enabled by the virtual social infrastructure, the factors such as community interaction, shared values, and common goals are still maintained, while participation from home has often been reflected on as the safe space by our participants. Lastly, as suggested by our mediation analysis, alternative means of group music-making still represented the context in which staff members displayed more positive behaviors, mediating the positive relationship between the two.

### Coping Through Virtual Group Music-Making

Creative music-making, and the community support enabled by youth participation in virtual music-making sessions essentially became a coping strategy for young participants in the early stages of COVID-19-related spatial distancing. Not only has engagement with music been associated with reduction in physiological and psychological stress (de Witte et al., 2020), it also offers a range of mechanisms facilitating individual as well as communal coping. Our research has demonstrated virtual music spaces allow individuals to utilize music as an emotion management tool through the psychological mechanisms of self-expression, as described by the Access-Awareness-Agency model (Saarikallio, 2019), and re-construct their “musical identities” and self-esteem. This is of special importance considering lower behavioral awareness was associated with greater general as well as peritraumatic distress in the context of COVID-19 pandemic (Kroska et al., 2020), and in light of identity challenges described by our young participants as well as professional adult musicians in the absence of musical opportunities in times of spatial distancing (Cohen and Ginsborg, 2021). Furthermore, social support by familiar as well as newly formed virtual music communities was also crucial, with research suggesting that the greater the number or strength of one's social groups, the easier coping with change was (Iyer et al., 2009; Steffens et al., 2021).

The significance of the availability of music groups in relation to inter- and intra-personal psychological experiences was also accentuated in the context of unavailability of school or other extracurricular opportunities. Emotional support of non-parental adult figures and peers has been recognized as valuable in supporting children and adolescents' psychosocial adjustment, above and beyond the support of their parents (Ryan et al., 1994; Demaray et al., 2009; Sterrett et al., 2011). In times of crisis, children and young people are particularly dependent on and susceptible to coping mechanisms of their parents or guardians (e.g., Earls et al., 2008; Diab et al., 2017), who battled with challenges of their own during the COVID-19 pandemic (e.g., Rajkumar, 2020). Further, considering a decrease in children's access to support since COVID-19 (e.g., Chen et al., 2020), and the raise in loneliness amongst schoolchildren (Loades et al., 2020), a safe space where one can re-connect with the self and others becomes an extraordinarily valued source of coping. However, it is important to recognize that not all young people opted in for virtual continuation of engagement with music groups, and therefore contribution of self-selection to the positive picture should also be acknowledged.

## Limitations and Future Directions

Firstly, we recognize methodological limitations to this study, as purposive sampling was utilized and we had little control over the development and collection of the secondary data sources, i.e., session reports, and surveys. There were a lot of unknown dependencies in the secondary data, especially with surveys treating each data entry as independent. We were unable to control for the cases when one young person attended more than one musical group, and in some cases, we were unable to match young people's responses to their parents', as surveys were sometimes distributed separately. Secondly, regarding the contribution to the understanding of the complex connection between group music-making and bonding, the indirect relationship between virtual group music-making and bonding via supporting staff behaviors should be further investigated in the context of in-person participation.

Moreover, while this paper illustrates heightened accessibility offered by virtual spaces for certain groups of young people (e.g., non-verbal, those from remote areas), it fails to acknowledge the challenges to virtual participation, as it does not involve those who chose not to participate in virtual group music-making alternatives, or were unable to. Considering research suggesting that some UK households with schoolchildren were only able to access the internet using a smartphone, or had little to no IT access at home, a challenge greater in areas of high eligibility to free school meals (written evidence from Just Fair, and Ask Research, as cited in House of Lords Covid-19 Committee, 2021), future research must address systemic inequalities in access to everyday, and virtual music education. Lastly, growing understanding of the use of virtual reality tools for re-enactments of virtual cultural experiences has a potential to replicate the experience of group music-making and contribute to a high sense of presence and togetherness (e.g., Bellini et al., 2018; Tarr et al., 2018). We believe such research is of special importance to development of future virtual or hybrid approaches to virtual music-making, especially considering the fight with COVID-19is far from over (Scudellari, 2020).

## Conclusion

In conclusion, virtual group music-making sessions were important for the coping of young musicians in times of spatial distancing during the COVID-19 pandemic, especially in the context of the lack of non-parental and peer support via schools. Development in intra-personal psychological outcomes, i.e., emotional competence and confidence, was promoted via creative self-expression, as well as staff support of their basic psychological needs, particularly for autonomy. While development on an inter-personal level was less profound, virtual music sessions provided communal support, and enabled preservation of musical communities via recollection of established communal features and connections, further supported via engagement with virtual alternatives to group music-making, mediated by supportive staff behaviors.

## Data Availability Statement

The raw data supporting the conclusions of this article will be made available by the corresponding author upon request, without undue reservation.

## Ethics Statement

The studies involving human participants were reviewed and approved by Sciences Technology Cross-Schools Research Ethics Committee, University of Sussex. The patients/participants provided their written informed consent to participate in this study.

## Author Contributions

ML reviewed the literature, formulated the study design, gained ethical approval, recruited participants, analyzed the data, and drafted the manuscript. RBar and KP assisted with session observations and contributed to observation checklist modifications. RBan supervised all of this work, and reviewed, edited, and finalized the manuscript with ML. All authors contributed to the article and approved the submitted version.

## Conflict of Interest

Some music projects involved with this research are funded and implemented by the organisation providing part of the research funding received. The authors declare that the research was conducted in the absence of any commercial or financial relationships that could be construed as a potential conflict of interest.

## Publisher's Note

All claims expressed in this article are solely those of the authors and do not necessarily represent those of their affiliated organizations, or those of the publisher, the editors and the reviewers. Any product that may be evaluated in this article, or claim that may be made by its manufacturer, is not guaranteed or endorsed by the publisher.

## Footnotes

^1^ https://www.theguardian.com/world/2020/mar/14/solidarity-balcony-singing-spreads-across-italy-during-lockdown

^2^ https://dancetabs.com/2020/09/outdoors-ballet-some-different-responses-to-lockdown-from-around-the-world/

^3^ https://www.gettyimages.com/detail/news-photo/people-look-at-a-photograph-by-slovenian-photographer-news-photo/1210189141

^4^https://www.bbc.com/news/av/uk-52860785.

^5^ https://www.aesthetics.mpg.de/en/research/department-of-music/musicovid-an-international-research-network.html

^6^This cannot reflect the total number of participants due to surveys being anonymous and obtained from various potentially overlapping sources (e.g., a young person attending more than one activity).

^7^Data aggregation process is described in the Analysis plan section of the paper, and in the Supplementary Materials.

^8^Qualitative and quantitative survey responses are treated separately, as not all surveys included the two together.

^9^Please refer to the Supplementary Materials for further details on the confirmatory factor analysis process, and specific questions allocated to each factor.

^10^Please refer to the Supplementary Materials for further details on the outcome variable aggregation process.

^11^Please refer to the Supplementary Materials for the contrasts table.

^12^Interval hypothesis testing was favored over the point null hypothesis as it allowed us to specify the interval of hypotheses rather than hypothesising specific mean differences (Heck et al., 2020). We performed one-tailed superiority analyses for the alternative hypothesis of mean difference being greater than zero against the null hypothesis of mean difference being zero or negative. Virtual setting was treated as the minuend in order to determine whether development took place in comparison to the preceding in-person setting.

^13^Region of practical equivalence (ROPE) was selected as 2.25–3.75 for session report scores, and 3.25–4.75 for survey scores.

^14^We utilized Bayesian estimations of credible intervals in order to simulate and plot posterior distributions and highest density intervals for the individual survey questions against the ROPE selected previously. This was performed using the R package “BayesianFirstAid,” based on the Bayesian Estimation Supersedes the t-Test (BEST) model developed by Kruschke (2013) and utilizing Just Another Gibbs Sampler (JAGS) program for Markov Chain Monte Carlo (MCMC) simulation, developed by Martyn Plummer.

^15^See Supplementary Materials for contrasts tables.

^16^Region of practical equivalence (ROPE) from −1.50 to 1.50 was selected as the score of 1 indicated only “*slight”* impact.

^17^Three basic psychological needs of the Self-determination theory are the need for autonomy (freedom in engagement and choices), competence (perceptions of ability to participate and contribute), and relatedness (feeling of connectedness with others involved; Deci and Ryan, 2000).
