# Supplementary material for: “It All Makes Us Feel Together”: Young People's Experiences of Virtual Group Music-Making During the COVID-19 Pandemic
Source: Front Psychol. 2021 Aug 5;12:703892. doi: 10.3389/fpsyg.2021.703892 (PMC8374080; doi:10.3389/fpsyg.2021.703892)
Supplement: Supplementary file 1 [file Data_Sheet_1.docx]

Supplementary Materials

# Supplementary Materials to the Methods Section

## List of Survey Questions Used by Music Education Hubs

| **SUPPLEMENTARY TABLE 1**  *A selection of all open-ended survey questions used by music education hubs involved, and quantitative questions used by all music education hubs.* | |
| --- | --- |
| **Survey type** | **List of questions** |
| Staff survey | 1. What was your experience of online teaching like? ^a^ 2. What do you think were young people’s experiences of having these online music lesson opportunities? Could you identify some benefits and some drawbacks of this? ^a^ 3. How was the experience of making music together online different from group music-making in person (please describe what you think the main differences were)? ^a^ 4. Do you have any suggestions for improvements and changes to be considered in planning for any future virtual music teaching if in-person music-making isn’t possible? ^a^ |
|  | 1. Positive Aspects - Please tell us what went well in the project? ^a^ 2. Any other comments about your experience and/or support needed to help you develop your practice? ^a^ |
|  | 1. Is there anything else you'd like to say about this student? (survey about individual young people) ^a^ |
| Parent survey | 1. Do you have any other comments or suggestions about new activities that you would like [Music education hub] to provide? ^a^ 2. What are your feelings overall regarding the virtual music learning opportunities during lockdown? Could you identify any benefits or drawbacks? ^a^ 3. Do you have any suggestions as to how online music tuition can be improved should we need to continue to offer this until in person tuition can resume? ^a^ |
|  | 1. We know that online engagement is not ideal for everyone. Please tell us what we can do to help make online activities work as well as possible for you/your child. (Session planning questionnaire) ^a^ |
|  | 1. Please let us know in your own words how the music lessons have helped your child/young person during the current health pandemic? ^a^ 2. Please let us know how this service could be improved? ^a^ 3. As a result of engaging with this virtual music-making session, my child's level of overall well-being has… (used by all) ^b^ 4. As a result of engaging with this virtual music-making session, my child's level of confidence has… (used by all) ^b^ 5. As a result of engaging with this virtual music-making session, my child's social skills have… (used by all) ^b^ 6. As a result of engaging with this virtual music-making session, my child's level of feeling calm and peaceful has… (used by all) ^b^ |
| Young people survey | 1. What did you like about the online music sessions? ^a^ 2. What would you improve or change for future online music teaching? ^a^ |
|  | 1. Is there anything else you'd like to say about the music project you took part in? ^a^ |
|  | 1. What do you enjoy most about online rehearsals? ^a^ 2. During lockdown, how important has the Virtual Music Centre been in helping you with your music-making? ^a^ 3. What would you change about these rehearsals, and why? Please give as much detail as possible. ^a^ 4. What would you like to add to weekly rehearsals, if you could? ^a^ |
|  | 1. Can you tell us about your music from sessions? ^a^ 2. What are you getting out of the sessions? ^a^ 3. If spaces were to stay online what should stay and what should change? 4. As a result of engaging with this virtual music-making session, my level of happiness has… (used by all) ^b^ 5. As a result of engaging with this virtual music-making session, my level of confidence has… (used by all) ^b^ 6. As a result of engaging with this virtual music-making session, my social skills have… (used by all) ^b^ 7. As a result of engaging with this virtual music-making session, my level of feeling calm and peaceful has… (used by all) ^b^ |
| - *Note: a = open-ended question, b = quantitative question with responses made on a scale: 7 got very much better, 6 got moderately better, 5 got slightly better, 4 not changed, 3 got slightly worse, 2 got moderately worse, 1 got very much worse.* | |

## Adjusted and Rephrased Observation Checklist

| **SUPPLEMENTARY TABLE 2**  *Behaviour items of the adjusted and rephrased observation checklist, adopted from Quested, Ntoumanis, Stenling, Thogersen-Ntoumani, and Hancox (2018).* | |
| --- | --- |
| **Behaviour category** | **Item** |
| Need-thwarting | 1. Using a language or a tone that is pressurising or could induce feelings of guilt or shame when communicating commands or goals ^a^ 2. Criticising, belittling, devaluing or dismissing participants ^a^ 3. Showing disregard or rejection for participants’ feelings, preferences, opinions and feedback ^a^ 4. Comparing participants against each other or being overly competitive ^a^ |
| Need-indifferent | 1. Providing unclear or confusing instructions without appropriate demonstrations ^b^ 2. Talking in ways that are motivationally “empty” (e.g., “keep going”, "cool", "okay"…) ^b^ 3. Giving instructions without expecting young people's responses ^b^ 4. Tutors chatting amongst themselves (e.g. solving technical difficulties, making jokes etc.) ^c^ |
| Need-supportive | 1. Tutors chatting with participants / telling participants about irrelevant topics ^c^ 2. Ensuring participants' understanding of instructions through encouraging questions and feedback (e.g. "Is that clear?", "Does anyone have any questions?" …) ^b^ 3. Giving meaningful, appropriate explanations and demonstrations ^b^ 4. Using an inclusive and considerate language (e.g., “we could try", "let's" ...) ^b^ 5. Acknowledging the participants’ comments / feelings and responding appropriately ^a^ 6. Offering direct or indirect meaningful praise which is unconditional (e.g. "well-done all") ^b^ 7. Providing young people with constructive and relevant feedback ^c^ 8. Create opportunities for participants' active participation and contribution to sessions (e.g. demonstrating, answering knowledge-related questions, asking what to do next…) ^b^ 9. Creating opportunities to interact with participants (e.g. addressing them, "who wants to do…") ^b^ |
| Music | 1. Group music making or turn-taking ^c^ |
| *Note: ^a^ = original item, ^b^ = original rephrased or adjusted item, ^c^* *= new item.* | |

## Observations Pilot and Rater Training Information

Three independent raters were involved with the observations, the first author (Rater 1) and the second and third authors (Rater 2 and Rater 3). Rater 1 observed all sessions in combination with either Rater 2 (42% of chunks observed) or Rater 3 (58% of chunks observed), based on their availability. Prior to data collection, Rater 1 attended three different virtual music activities in order to familiarise themselves with the session set-up and dynamics, which informed the planning and adjustment of the observation materials. This was followed by a training period for Raters 2 and 3, led by Rater 1 for each rater separately. The training followed the relevant procedure steps (1-4) adopted by Quested and colleagues (2018), excluding the steps training for independent coding, which was not implemented by this project (step 5 and above). Specifically, raters were asked to read the papers contributing to the design of the study (Levstek & Banerjee, 2021; Quested et al., 2018) and a summary document containing theoretical and practical background information on SDT (based on Deci & Ryan, 1990, 2000). Moreover, raters were asked to familiarise themselves with an adopted version of a practical guide document kindly shared by Dr Quested, which included coding instructions, item descriptions, intensity scoring guide and an example coding template. This was followed by a Zoom call covering step 2 and step 3 of the training procedure, including a discussion of the theoretical background and an introduction to the coding tool and process. Once both raters were familiarised with SDT and the coding process, they attended three virtual music sessions in order to pilot the adjusted materials and practice collaborative coding activity with Rater 1, representing the final step of the training process. The piloting enabled raters to discuss adjusted coding materials and ensure unanimous understanding of items, as well as develop a convenient communication and time-keeping method to be adopted during the duration of virtual music sessions. The raters agreed for the Rater 1 to act as a timekeeper and stay connected with the other rater via a separate ongoing phone call in order to sync the two-minute window change, 15-minute chunk change, and to discuss the intensity ratings at the end of each 12-minute frequency rating period.

## Inter-Rater Reliability for Observation Data

All sessions were observed by Rater 1 (the first author) in combination with either Rater 2 or Rater 3 (the second and third authors). Therefore, inter-rater reliability for Rater 1 with either of the two raters was calculated in a categorical as well as continuous format. Inter-rater reliability was calculated for all items together, as well as for separate behaviour types, need thwarting, need indifferent and need supportive. Group music-making behaviour was not included in calculations as these were only recorded by Rater 1. The inter-rater reliability in a categorical format was calculated for each 2-minute window of each item recorded for all 15-minute chunks, examining the agreement for raters in recording behaviours or not. With this format, we have encountered one of the paradoxes of Cohen’s kappa value, when a low value occurs at an especially high agreement (Feinstein & Cicchetti, 1990, as cited in Yilmaz & Saracbasi, 2017). This issue was especially apparent when coding for thwarting behaviours as the category frequencies were unequal due to rare occurrence of need thwarting behaviours. Various Monte Carlo simulations (e.g. Quarfoot & Levine, 2016; Tran, Dolgun, & Demirhan, 2020) have deemed Gwet’s AC1 (K. L. Gwet, 2014) and Brennan-Prediger’s kappa (Brennan & Prediger, 1981) as more appropriate measures of categorical inter-rater reliability due to the use of a more conservative approach in adjusting for chance. Function ‘bp.coeff.raw’ was used for Brennan-Prediger’s kappa calculation, and ‘gwet.ac1.raw’ for Gwet’s AC1 from the package ‘irrCAC’ (Kilem L. Gwet, 2019).

The inter-rater reliability in a continuous format was calculated for the total number of windows recording for each item for all 15-minute chunks, examining whether the raters recoded for the same number of behaviour occurrences within a chunk, even if not recorded for in the same 2-minute window. This method of inter-rater reliability calculation was deemed preferable due to the fact that the changes between 2-minute windows were monitored by Rater 1 and communicated to the other rater via the phone, making it possible for the recording of behaviours taking place at the change of the two windows to be allocated to different windows by the two raters. One-way intraclass correlation coefficient was chosen for calculating inter-rater reliability of the continuous format for Rater 1 with either of the two rater, using a ‘icc’ function of the package ‘irr’ (Gamer, Lemon, & Fellows Puspendra Singh, 2019).

| **SUPPLEMENTARY TABLE 3**  *Inter-rater reliability scores for observation tallies.* | | | | |
| --- | --- | --- | --- | --- |
|  | **Categorical** | | | **Continuous** |
|  | P(a) | Brennan & Prediger's Kappa | Gwet’s AC1 | ICC |
| All items (17) | .83 | .66 | .72 | .86 |
| Need thwarting items (4) | .98 | .96 | .98 | .67 |
| Need indifferent items (4) | .83 | .65 | .75 | .74 |
| Need supporting items (9) | .77 | .54 | .54 | .80 |
| *Note: P(a) = the percent of agreement, ICC = intraclass correlation coefficient.* | | | | |

## Data Reduction and Aggregation

### Session Report and Survey Data

All variables were aggregated by calculating the mean score of the corresponding items. Confirmatory factor analysis was performed on the session report data (R package ‘lavaan’; Rosseel, 2012). Informed by outcome classification in previous research (Levstek & Banerjee, 2021), two-factor solution included ‘Intra-personal outcomes’ variable (items: “*How many of the YP displayed feelings of general well-being as a result of engagement with music: All or nearly all / more than a half / about a half / less than a half / none or just a few”*, and “*How many of the YP displayed feelings of general self-efficacy and self-esteem, as a result of engagement with music: All or nearly all / more than a half / about a half / less than a half / none or just a few”*) and ‘Inter-personal outcomes’ variable (items: “*Overall, YP’s attitude towards each other was very good / quite good / sufficient / quite bad / very bad*”; “*Overall, YP’s attitude to others at the venue was very good / quite good / sufficient / quite bad / very bad*”; and “*Overall, YP’s communication with each other was very good / quite good / sufficient / quite bad / very bad”),* as represented in Table 4. Based on the model fit indices and specified research hypotheses, two-factor solution was deemed preferable.

For survey data, the item representing change in social skills (*“As a result of engaging with this virtual music-making session, my / my child’s social skills have*”) was kept on its own in order to represent an inter-personal outcome dimension. Items representing change in well-being (“*As a result of engaging with this virtual music-making session, my level of happiness / my child’s level of overall well-being has 7 got very much better, 6 got moderately better, 5 got slightly better, 4 not changed, 3 got slightly worse, 2 got moderately worse, 1 got very much worse*”), calmness (*“As a result of engaging with this virtual music-making session, my / my child’s level of feeling calm and peaceful has 7 got very much better, 6 got moderately better, 5 got slightly better, 4 not changed, 3 got slightly worse, 2 got moderately worse, 1 got very much worse*”), and confidence (“*As a result of engaging with this virtual music-making session, my / my child’s level of confidence has 7 got very much better, 6 got moderately better, 5 got slightly better, 4 not changed, 3 got slightly worse, 2 got moderately worse, 1 got very much worse*”) were grouped in order to represent an intra-personal outcome dimension, with a satisfactory Cronbach’s alpha of .78.

| **SUPPLEMENTARY TABLE 4**  *Confirmatory factor analysis statistics for session report data.* | | | | | |
| --- | --- | --- | --- | --- | --- |
|  | **One factor** | **Two factors** | | |  |
|  | **Outcomes** | | **Intra-personal outcomes** | **Inter-personal outcomes** |  |
| **Items** |  | |  |  |  |
| Well-being | .58 | | .90 |  |  |
| Confidence | .52 | | .77 |  |  |
| Communication | .50 | |  | .50 |  |
| Attitude | .88 | |  | .90 |  |
| Attitude wide | .82 | |  | .82 |  |
| **Alpha** | .80 | | .81 | .78 |  |
| **Model fit** |  | |  |  |  |
| Chi-sq | 54.14 | | 1.98 |  |  |
| df | 5 | | 4 |  |  |
| p-value | .00 | | .74 |  |  |
| CFI | .80 | | 1 |  |  |
| RMSEA | .28 | | 0 |  |  |
| BIC | 1178.47 | | 1131.17 |  |  |
| *Note: Alpha = Cronbach’s alpha for item groupings, Chi-sq = chi-square, df = chi-square degrees of freedom, p-value = chi-square p-value, CFI =* *comparative fit index, RMSEA = root mean square error of approximation, BIC = Bayesian information criterion.* | | | | | |

### Observation Data

The need-relevant instructor behaviours scale aggregation process suggested by Quested, Ntoumanis, Stenling, Thogersen-Ntoumani, and Hancox (2018) was adopted and adjusted in order to suit our modifications. The following process is represented with equations 1.a to 2.a below, with ‘n’ representing a total number of cases, ‘x’ and ‘y’ representing independent raters, ‘a’ representing a behaviour type (need-supporting, need-indifferent, or need-thwarting), and ‘k’, ‘l’, ‘n’ representing individual checklist behaviour items.

We firstly created sum variables for each question’s tallies, indicating number of event occurrence per chunk by adding together individual window values (e.g. for question ‘k’ which represented a behaviour type ‘a’ by rater ‘x’, see Equation 1.a). Following this, we created overall tally scores for all three types of need-related behaviours (need-thwarting, need-indifferent and need-supportive) by averaging appropriate question (e.g. for behaviour type ‘a’ by rater ‘x’, see Equation 1.b). We decided to average rather than sum behaviour type scores as there is a different a number of questions for need-supportive type of behaviour (9) than for need-thwarting (4) and need-indifferent (4) category. This aggregation step was not necessary for the group music-making tally as it was one item. Those aggregated behaviour tally scores were then averaged between the rater pairs in order to provide one score per observation chunk (e.g. behaviour type ‘a’ average tally scores by tater ‘x’ and rater ‘y’, see Example 1.c).

Need impact rating variables were aggregated by calculating overall need impact rating scores for the separate three basic psychological needs as a difference between the supporting and thwarting values (see Example 2.a). Note that the aggregated variable can now range from 3 to -3, depending on whether need supporting or need thwarting scores are prevailing. Averaging by the rater was not necessary as these scores were agreed upon a discussion after each 15-minute chunk observation period.

**EQUATIONS 1.A TO 1.C**

*Behaviour tally aggregation equations.*

$$1.a) Behaviour type a item k rater x tally sum score = Behaviour type a behaviour item k rater x window 1 tally score + Behaviour type a behaviour item k rater x window 2 tally score +\ldots+ Behaviour type a behaviour item k rater x window n tally score$$

$$1.b) Behaviour type a rater x average tally score = (Behaviour type a item k rater x tally sum score +Behaviour type a item l rater x tally sum score + \ldots+ Behaviour type a item n rater x tally sum score) / n$$

$$1.c) Behaviour type a overall tally score= (Behaviour type a rater x average tally score + Behaviour type a rater y average tally score ) / 2$$

**EQUATIONS 2**

*Basic psychological need rate score equation.*

$$2.a) Basic psychological need a overall impact rate score=Basic psychological need a supporting impact rate score-Basic psychological need a thwarting impact rate score$$

# Supplementary Materials to the Results Section

## Preliminary Descriptive Tables and Correlation Matrices

### Session Report Data

| **SUPPLEMENTARY TABLE 5**  *Descriptive statistics for quantitative session report variables.* | | | | | | | | |
| --- | --- | --- | --- | --- | --- | --- | --- | --- |
| **Variables** | **Mean** | **SD** | **N** | **N missing (percentage)** | **Mean virtual only** | **SD virtual only** | **N virtual only** | **N missing (percentage) virtual only** |
| **Session information** |  |  |  |  |  |  |  |  |
| Session type ^b^ |  |  | 142 | 0 (0%) |  |  | 56 | 0 (0%) |
| - Mainstream ensembles | -- | -- | 0 | -- | -- | -- | 0 | -- |
| - Inclusive ensembles | .37 |  | 52 |  | .20 |  | 11 |  |
| - Music spaces | .63 |  | 90 |  | .80 |  | 45 |  |
| Venue ^b^ |  |  | 142 | 0 (0%) |  |  |  |  |
| - In-person | .61 |  | 86 |  | -- | -- | -- | -- |
| - Virtual | .39 |  | 56 |  | 1 |  | 56 | 0 (0%) |
| **Survey items** |  |  |  |  |  |  |  |  |
| Well-being   (1 -5) ^a^ | 4.62 | 0.68 | 142 | 0 (0%) | 4.80 | 0.48 | 56 | 0 (0%) |
| Confidence   (1 – 5) ^a^ | 4.41 | 0.78 | 142 | 0 (0%) | 4.71 | 0.49 | 56 | 0 (0%) |
| Communication  skills (1 – 5) ^a^ | 4.38 | 0.68 | 142 | 0 (0%) | 4.29 | 0.76 | 56 | 0 (0%) |
| Attitude (1 – 5) ^a^ | 4.63 | 0.61 | 142 | 0 (0%) | 4.68 | 0.58 | 56 | 0 (0%) |
| Attitude wide  (1 – 5) ^a^ | 4.65 | 0.63 | 129 | 13 (9%) | 4.74 | 0.53 | 50 | 6 (11%) |
| *Notes: Means for categorical data represent case percentages for its levels; a = continuous variables, b = categorical variables.* | | | | | | | | |

| **SUPPLEMENTARY TABLE 6**  *Correlation matrix for the session report continuous variables when filtered for virtual setting only.* | | | | | | |
| --- | --- | --- | --- | --- | --- | --- |
|  | **Well-being** | **Confidence** | **Communication** | **Attitude** | **Attitude wide** |  |
| **Well-being** | 1 |  |  |  |  |  |
| **Confidence** | .75*** | 1 |  |  |  |  |
| **Communication** | .21 | .32* | 1 |  |  |  |
| **Attitude** | .42** | .31* | .38** | 1 |  |  |
| **Attitude wide** | .45*** | .32* | .43** | .84*** | 1 |  |
| *Note: * p < .05, ** p < .01, *** p < .001.* | | | | | | |

### Survey Data

| **SUPPLEMENTARY TABLE 7**  *Descriptive statistics for quantitative survey data variables.* | | | | |
| --- | --- | --- | --- | --- |
| **Variables** | **Mean** | **SD** | **N** | **N missing (percentage)** |
| **Participant information** |  |  |  |  |
| Session type ^b^ |  |  | 96 | 0 (0%) |
| - Ensembles | .72 |  | 69 |  |
| - Inclusive ensembles | .21 |  | 20 |  |
| - Music spaces | .07 |  | 7 |  |
| Subject ^b^ |  |  | 96 | 0 (0%) |
| - Young person | .68 |  | 65 |  |
| - Parent | .32 |  | 31 |  |
| **Survey items** |  |  |  |  |
| Well-being / Happiness (1 -7) ^a^ | 5.39 | 1.20 | 96 | 0 (0%) |
| Feeling calm and peaceful (1 – 7) ^a^ | 4.76 | 1.21 | 96 | 0 (0%) |
| Confidence (1 – 7) ^a^ | 5.32 | 1.22 | 96 | 0 (0%) |
| Social skills (1 – 7) ^a^ | 4.73 | 1.08 | 96 | 0 (0%) |
| *Notes: Means for categorical data represent case percentages for its levels; a = continuous variables, b = categorical variables.* | | | | |

| **SUPPLEMENTARY TABLE 8**  *Correlation matrix for the continuous survey variables.* | | | | |
| --- | --- | --- | --- | --- |
|  | **Well-being** | **Calmness** | **Confidence** | **Social skills** |
| **Well-being** | 1 |  |  |  |
| **Calmness** | .58*** | 1 |  |  |
| **Confidence** | .53*** | .40*** | 1 |  |
| **Social skills** | .57*** | .47*** | .55*** | 1 |
| *Note: * p < .05, ** p < .01, *** p < .001.* | | | | |

### Observations Data

| **SUPPLEMENTARY TABLE 9**  *Descriptive statistics for observation data variables.* | | | | |
| --- | --- | --- | --- | --- |
| **Variables** | **Mean** | **SD** | **N** | **N missing (percentage)** |
| **Session information** |  |  |  |  |
| Session type ^b^ |  |  | 59 | 0 (0%) |
| - Mainstream ensembles | .64 | -- | 38 |  |
| - Inclusive ensembles | -- | -- | -- | -- |
| - Music spaces | .36 | -- | 21 |  |
| Day ^b^ |  |  | 59 | 0 (0%) |
| - First observation of the session | .51 | -- | 30 |  |
| - Second observation of the session | .49 | -- | 29 |  |
| Chunk ^b^ |  |  | 59 | 0 (0%) |
| - Chunk 1 – first set of 15 minutes | .27 | -- | 16 |  |
| - Chunk 2 – second set of 15 minutes | .27 | -- | 16 |  |
| - Chunk 3 – third set of 15 minutes | .25 | -- | 15 |  |
| - Chunk 4 – last set of 15 minutes | .20 | -- | 12 |  |
| Observer pair ^b^ |  |  | 59 | 0 (0%) |
| - Rater 1 and Rater 2 | .42 |  | 25 |  |
| - Rater 1 and Rater 3 | .58 |  | 34 |  |
| **Tally variables** |  |  |  |  |
| Need-thwarting tally score (0 -6) ^a(agg)^ | 0.11 | 0.18 | 59 | 0 (0%) |
| Need-indifferent tally score (0 – 6) ^a(agg)^ | 1.15 | 0.52 | 59 | 0 (0%) |
| Need-supportive tally score (0 – 6) ^a(agg)^ | 2.61 | 0.67 | 59 | 0 (0%) |
| Group music-making tally score (0 – 6) ^a(agg)^ | 1.69 | 1.66 | 59 | 0 (0%) |
| **Staff rated need support variables** |  |  |  |  |
| Overall autonomy impact score (-3 – 3) ^a(agg)^ | 2.43 | 0.58 | 59 | 0 (0%) |
| Overall competence impact score (-3 – 3) ^a(agg)^ | 1.93 | 0.75 | 59 | 0 (0%) |
| Overall relatedness impact score (-3 – 3) ^a(agg)^ | 2.19 | 0.77 | 59 | 0 (0%) |
| *Notes: Means for categorical variables represent case percentages for its levels; a = continuous variables, b = categorical variables, agg = aggregated variables.* | | | | |

| **SUPPLEMENTARY TABLE 10**  *Correlation matrix for the continuous survey variables.* | | | | | | | |
| --- | --- | --- | --- | --- | --- | --- | --- |
|  | **Need-thwarting tally** | **Need-indifferent tally** | **Need-supporting tally** | **Group music-making tally** | **Autonomy rate** | **Competence rate** | **Relatedness rate** |
| **Need-thwarting tally** | 1 |  |  |  |  |  |  |
| **Need-indifferent tally** | .21 | 1 |  |  |  |  |  |
| **Need-supporting tally** | .09 | .16 | 1 |  |  |  |  |
| **Group music-making tally** | .42** | .01 | .45*** | 1 |  |  |  |
| **Autonomy rate** | -.03 | .01 | .05 | .14 | 1 |  |  |
| **Competence rate** | -.37** | -.08 | .44*** | .18 | .06 | 1 |  |
| **Relatedness rate** | -.10 | .17 | .70*** | .23(*) | .12 | .51*** | 1 |
| *Note: * p < .05, ** p < .01, *** p < .001.* | | | | | | | |

## Analysis of Variance Contrasts Tables

See tables below for orthogonal contrasts selected for categorical variables with more than two levels.

| **SUPPLEMENTARY TABLE 11**  *Contrasts for survey data variable session type.* | | |
| --- | --- | --- |
|  | **Inclusive vs mainstream sessions** | **Ensembles vs music spaces** |
| Ensembles | -2 | 0 |
| Inclusive ensembles | 1 | 1 |
| Music spaces | 1 | -1 |

| **SUPPLEMENTARY TABLE 12**  *Contrasts for observation data variable observation chunks.* | | | |
| --- | --- | --- | --- |
|  | **First vs second half** | **Chunk 1 vs chunk 2** | **Chunk 3 vs chunk 4** |
| Chunk 1 | 1 | 1 | 0 |
| Chunk 2 | 1 | -1 | 0 |
| Chunk 3 | -1 | 0 | 1 |
| Chunk 4 | -1 | 0 | -1 |

| **SUPPLEMENTARY TABLE 13**  *Contrasts for the long observation data variable basic psychological need type.* | | |
| --- | --- | --- |
|  | **Autonomy and competence vs relatedness** | **Autonomy vs competence** |
| Autonomy impact rate | 1 | 1 |
| Competence impact rate | 1 | -1 |
| Relatedness impact rate | -2 | 0 |

# References Included in the Supplementary Materials

Brennan, R. L., & Prediger, D. J. (1981). Coefficient Kappa: some uses, misuses, and alternatives. *Educational and Psychological Measurement*, *41*(3), 687–699.

Deci, E. L., & Ryan, R. M. (1990). A motivational approach to self: Integration in personality. *Nebraska Symposium on Motivation: Perspectives on Motivation*, *38*, 237–288. https://doi.org/10.1207/s15326985ep2603&4_6

Deci, E. L., & Ryan, R. M. (2000). The “What” and “Why” of goal pursuits: Human needs and the self-determination of behavior. *Psychological Inquiry*, *11*(4), 227–268. https://doi.org/10.1207/S15327965PLI1104_01

Gamer, M., Lemon, J., & Fellows Puspendra Singh, I. (2019). irr: Various Coefficients of Interrater Reliability and Agreement. *R Package Version 0.84.1*. Retrieved from https://cran.r-project.org/package=irr

Gwet, K. L. (2014). *Handbook of inter-rater reliability: The definitive guide to measuring the extent of agreement among raters*. MD: Advanced Analytics, LLC.

Gwet, Kilem L. (2019). irrCAC: Computing Chance-Corrected Agreement Coefficients (CAC). *R Package Version 1.0.* Retrieved from https://cran.r-project.org/package=irrCAC

Levstek, M., & Banerjee, R. (2021). A model of psychological mechanisms of inclusive music-making: Empowerment of marginalised young people. *[Submitted for Publication]*.

Quarfoot, D., & Levine, R. A. (2016). How Robust Are Multirater Interrater Reliability Indices to Changes in Frequency Distribution? *American Statistician*, *70*(4), 373–384. https://doi.org/10.1080/00031305.2016.1141708

Quested, E., Ntoumanis, N., Stenling, A., Thogersen-Ntoumani, C., & Hancox, J. E. (2018). The Need-Relevant Instructor Behaviors Scale: Development and initial validation. *Journal of Sport and Exercise Psychology*, *40*(5), 259–268. https://doi.org/10.1123/jsep.2018-0043

Rosseel, Y. (2012). Lavaan: An R package for structural equation modeling. *Journal of Statistical Software*, *48*(2), 1–36. Retrieved from http://www.jstatsoft.org/v48/i02/

Tran, D., Dolgun, A., & Demirhan, H. (2020). Weighted inter-rater agreement measures for ordinal outcomes. *Communications in Statistics - Simulation and Computation*, *49*(4), 989–1003. https://doi.org/10.1080/03610918.2018.1490428

Yilmaz, A. E., & Saracbasi, T. (2017). Assessing agreement between raters from the point of coefficients and log-linear models. *Journal of Data Science*, *15*(1), 1–24. https://doi.org/10.6339/JDS.201701_15(1).0001
